# Strongly Anisotropic and Rapid Excitons Transport in Organic Crystal

**DOI:** 10.1002/advs.77660

**Published:** 2026-09-08

**Authors:** Minjie Li, Zheng‐Fei Liu, Jiajia Ma, Dayujia Huo, Ganglong Cui, Qing‐Zheng Yang, Yan Wan

**Affiliations:** ^1^ College of Chemistry Beijing Normal University Beijing People's Republic of China

**Keywords:** energy transfer, exciton transport, imaging, organic semiconductors, time‐resolved spectroscopy

## Abstract

Exciton diffusion plays a crucial role in the photoelectric conversion process. The increased diffusion length is beneficial to simplify device design and enhance photoelectric conversion efficiency. The isotropic exciton diffusion may lead to reduced transport efficiency directed to the charge separation site. Therefore, it's not only needed to increase the transport rate of excitons, but also needed to restrict the transport direction of excitons. Here we have successfully achieved strongly anisotropic and rapid excitons transport in thienopyrrole‐dicarbonyl fluoroboron organic crystal. Two organic crystals of SNH‐FB and SNEt‐FB have been synthesized to control the exciton transport rate and directions. The transient absorption imaging results indicate that no obvious exciton diffusion is observed in SNH‐FB crystals, but ethyl substituted SNEt‐FB crystals have a significantly enhanced exciton diffusion constant (∼2 cm^2^/s) along only one axis on the ab plane of the crystal. Theoretical calculations demonstrate that the coupling strength of molecules along different directions of the SNEt‐FB crystals is significantly different, making important contributions to the obvious anisotropy of exciton transport.

## Graphical abstract

The increased exciton diffusion length is beneficial to simplify device design and enhance photoelectric conversion efficiency. But the isotropic exciton diffusion may lead to reduced transport efficiency directed to the charge separation site. Here we have successfully achieved strongly anisotropic and rapid excitons transport in thienopyrrole‐dicarbonyl fluoroboron organic crystal.

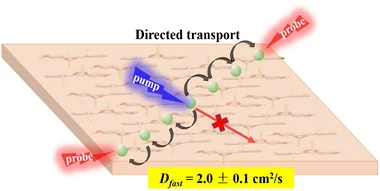

## 1 Introduction

Exciton transport is one of the key photophysical process, which is of great significance for light harvesting and charge separation in photovoltaic applications [1, 2, 3]. The limited mobility of excitons is frequently the primary factor constraining device performance, particularly in the case of organic solar cells (OSCs) [4, 5, 6]. Improving exciton transport rate is one of the effective means to increase the exciton diffusion length [7, 8]. The increased diffusion length allows for the utilization of a thicker photoactive layer, thereby streamlining device design and enhancing photoelectric conversion efficiency [8, 9, 10, 11, 12, 13, 14].

Exciton transport in organic polycrystalline materials is generally isotropic because the orientation of grains is randomly distributed [15, 16, 17, 18, 19]. But the core challenge in exciton‐based devices lies in establishing efficient exciton transport pathway to enhance charge separation efficiency, whereas isotropic exciton diffusion may lead to reduced transport efficiency directed to the charge separation site [20, 21, 22, 23]. Therefore, it's not only needed to increase the transport rate of excitons, but also needed to restrict the transport direction of excitons.

The investigation for directional transport is currently focusing on quasi‐one‐dimensional structures [24], such as nanofibers, but disorder in such systems usually hinders long‐range transport of excitons [25]. It's demonstrated that supramolecular aggregates can form weakly coupled one‐dimensional aggregated chains that enabling highly anisotropic exciton transport under low‐temperature conditions [4, 26]. There is also a class of molecular structures of the donor‐acceptor type, where the resulting crystal stacking induces strong intermolecular interactions leading to significant anisotropy, which in turn leads to strongly directed transport of excitons [15, 27]. However, the realization of strongly directed exciton transport in single‐component organic crystals is still difficult.

**FIGURE 1:**
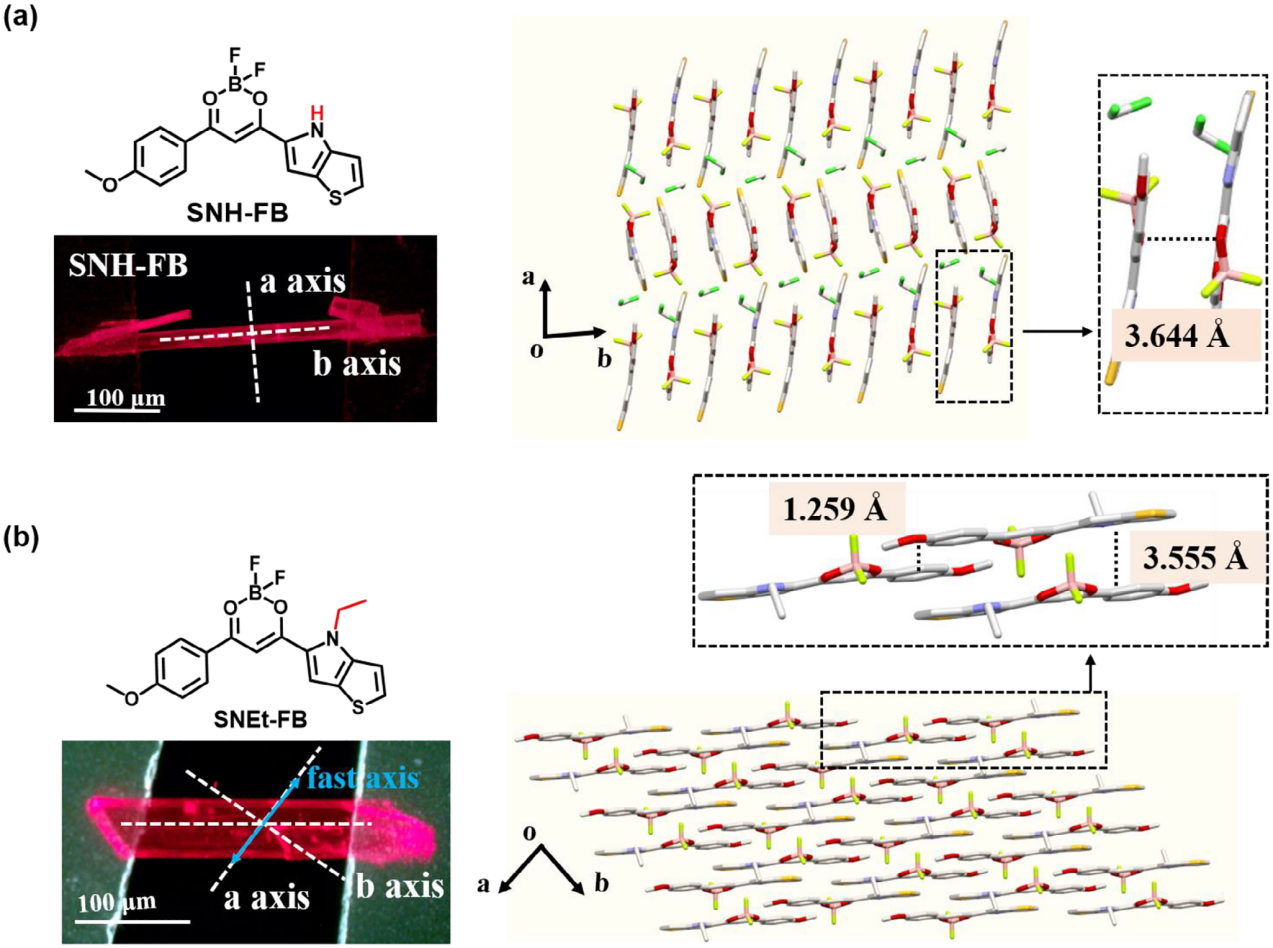
Molecular structure, photoluminescence microscopy image, and crystal structure of (a) SNH‐FB and (b) SNEt‐FB. The crystal axis and fast transport axis are labeled.

Here we have successfully achieved strongly anisotropic and rapid excitons transport on a plane of an organic crystal. Two thienopyrrole‐dicarbonyl fluoroboron organic crystals (SNH‐FB and SNEt‐FB, with molecular and crystal structures shown in Figure 1) have been synthesized to control the exciton transport rate and directions. The transient absorption microscopy (TAM) was used to directly visualize the exciton transport process in organic crystals. The results indicate that no obvious exciton diffusion is observed in SNH‐FB crystals. But ethyl substituted SNEt‐FB crystals have significantly enhanced exciton transport rate (diffusion constant *D* ∼ 2 cm^2^/s) along only one axis on the ab plane of the crystal. Theoretical calculations prove that such a strong anisotropic exciton transport mainly originates from the significant differences in the coupling strength between molecules along different directions.

## 2 Results and Discussion

### 2.1 Synthesis and Characterization

The synthesis steps, NMR and Mass spectra of SNH‐FB and SNEt‐FB molecules are presented in Supporting Information (Figures S1–S10). Single crystals of SNH‐FB and SNEt‐FB were obtained by slow evaporation of their solution in mixed solvents of dichloromethane and *n*‐hexane (v/v = 2:1). Crystal data and structure refinement of them are shown in Tables S1 and S2. Figure 1 illustrates the photoluminescence (PL) imaging and crystal structure of the two crystals utilized in optical measurements. The molecule 4‐(4H‐thieno[3,2‐b]pyrrole‐5‐yl)‐6‐(4‐methoxyphenyl)‐2,2‐difluoro‐2H‐1,3,2‐dioxaborinin‐1‐ium‐2‐uide (Figure 1a, abbreviated as SNH‐FB) is crystalline in orthorhombic system, cell parameters are *a* = 21.1573(2) Å, *b* = 7.98740(8) Å, *c* = 21.2600(2) Å, *α* = *β* = *γ* = 90°. The SNH‐FB crystals exhibit growth along the b‐axis, characterized by antiparallel molecular pairs that interlace with one another. The molecules are arranged end‐to‐end along the a‐axis and separated by the solvent molecule of dichloromethane. The incorporation of solvent molecules increases the distance between molecules and hinders the close packing, which should be adverse to exciton transport. The molecule 4‐(4‐ethyl‐thieno[3,2‐b]pyrrole‐5‐yl)‐6‐(4‐methoxyphenyl)‐2,2‐difluoro‐2H‐1,3,2‐dioxaborinin‐1‐ium‐2‐uide (Figure 1b, abbreviated as SNEt‐FB) is crystalline in triclinic system, cell parameters are *a* = 8.94545(10) Å, *b* = 8.98505(12) Å, *c* = 32.5232(3) Å, *α* = 83.5041(9)°, *β* = 89.0320(9)°, *γ* = 75.6694(11)°. In the SNEt‐FB crystal, a similar arrangement of antiparallel molecular pairs is observed along the b‐axis, akin to the SNH‐FB structure. However, a notable distinction of the SNEt‐FB crystal is the denser packing of antiparallel pairs along the a‐axis, leading to a more ordered arrangement that may be beneficial to exciton transport. The crystallographic orientations of the crystals were determined by indexing their SAED patterns using the unit‐cell parameters obtained from single‐crystal X‐ray diffraction (Figures S11 and S12).

### 2.2 Steady‐State Absorption and Photoluminescence Spectra

**FIGURE 2:**
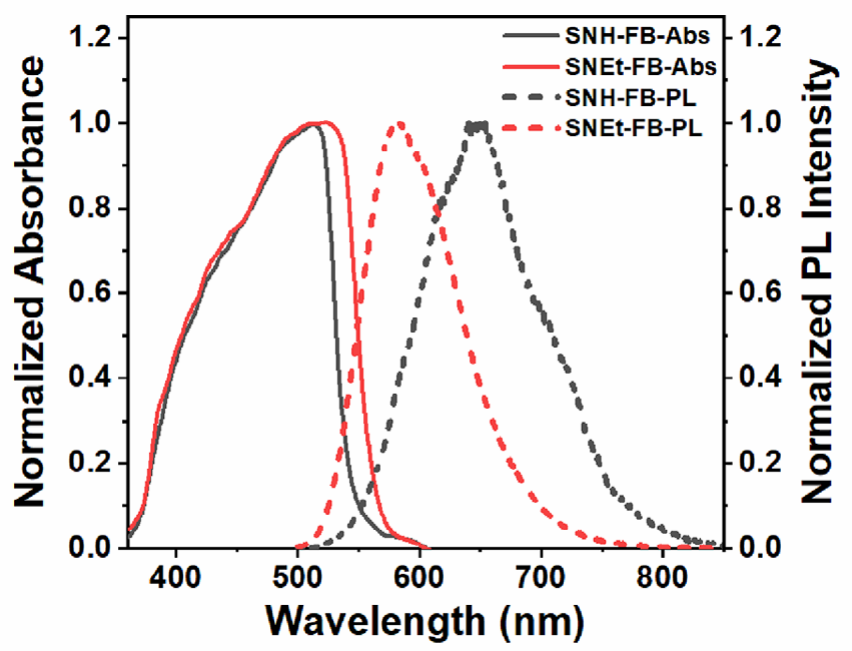
Normalized absorption (solid lines) and PL spectra (dashed lines, λ_ex_ = 453 nm) of SNH‐FB and SNEt‐FB crystals.

Figure 2 shows the steady‐state absorption and PL spectra of the two crystals. It's observed that both crystals exhibit broad absorption peaks in the range of 360–600 nm. The absorption peak of the SNH‐FB crystals is at 510 nm and that of the ethyl‐substituted SNEt‐FB crystal red‐shifts to 520 nm. When excited at 453 nm, the peak wavelengths of the PL spectra of SNH‐FB and SNEt‐FB crystals are ∼ 645 nm and ∼ 585 nm, respectively. It's clear that the introducing of ethyl group causes a blue‐shift of the PL band (while a red‐shift occurs for the absorption band), which has significantly reduced the Stokes shift. This results in a substantial overlap between the absorption and photoluminescence spectra (the overlap integral of SNEt‐FB is about six times of that of SNH‐FB), facilitating the energy transfer process between molecules [28].

### 2.3 Femtosecond Transient Absorption Spectroscopy

**FIGURE 3:**
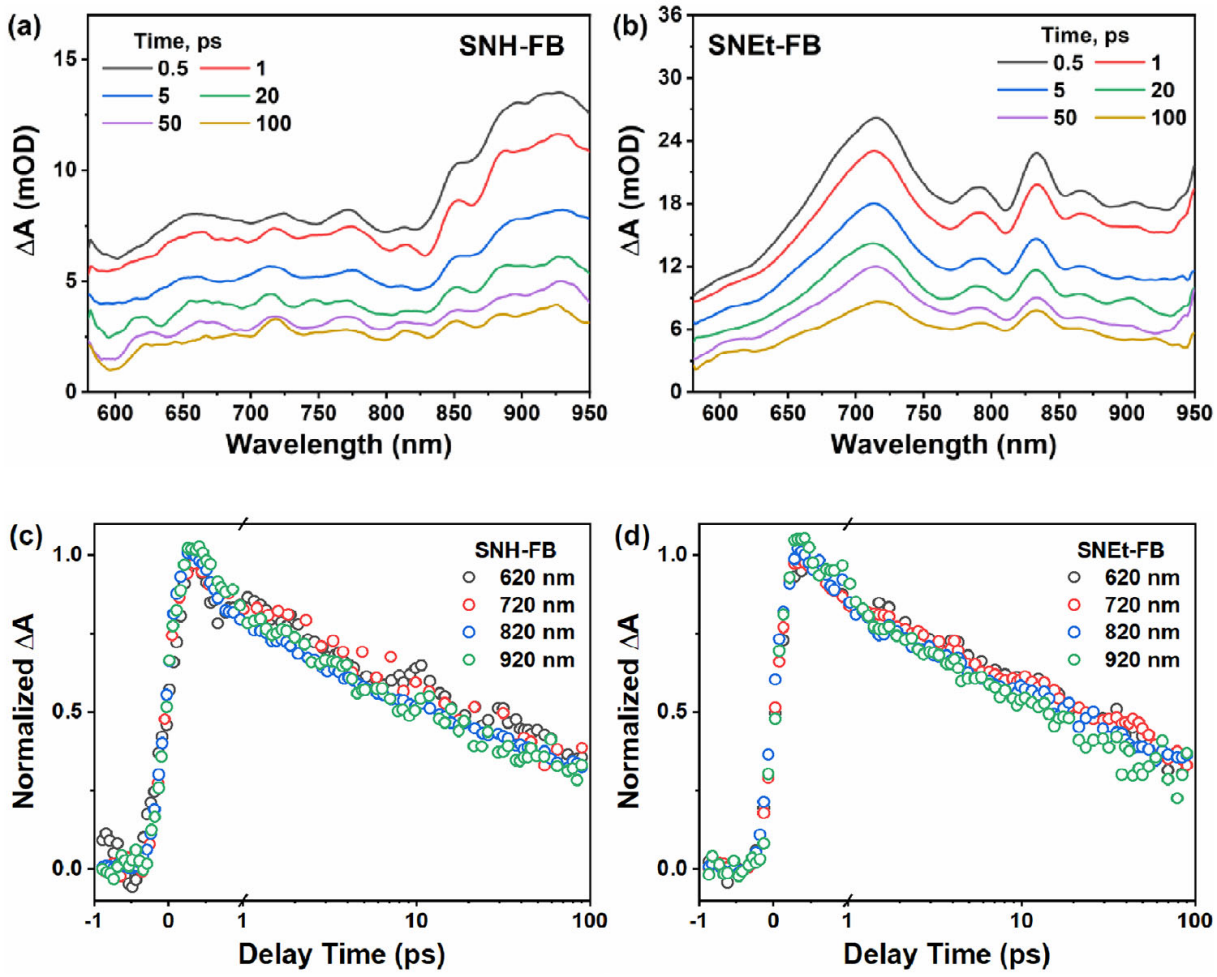
(a,b) The TA spectra of SNH‐FB and SNEt‐FB crystals at different delay times under 400 nm excitation. The polarization of the pump and probe light was parallel to the long axis of the crystals. (c,d) The TA dynamics at different probe wavelengths of SNH‐FB and SNEt‐FB crystals.

The femtosecond transient absorption (TA) spectra of single SNH‐FB and SNEt‐FB crystals have been measured to provide the necessary excited absorption information for subsequent TAM experiments. Figure 3a,b show the TA spectra of the two crystals, and both exhibit a wide excited state absorption (ESA) band (580–950 nm). The ESA signal reaches maximum at 0.5 ps and then slowly decays with delay time. The dynamics at different probe wavelengths are the same (Figure 3c,d), indicating that the wide ESA band is from the same excited state.

The TA spectra of the two crystals were also measured under varying pump and probe polarization, as shown in Figures S13–S16. The spectral characteristics and evolution remain the similarity, and the dynamics at characteristic wavelengths exhibits a consistency, indicating that the TA dynamics of the two crystals are not polarization sensitive.

### 2.4 Direct Imaging of Long‐Range Exciton Transport by Using TAM

The pump wavelength of 400 nm and the probe wavelength of 820 nm (to avoid the interference of PL signal by using a long pass filter at 800 nm) were selected for the TAM measurements, based on the TA and PL spectra. Figures S17 and S18 show a comparison of exciton dynamics of the two crystals under different pump and probe polarization. The exciton dynamics remains the same, which is consistent with the results of the TA spectra. Under higher excitation density, multiple excitons usually interact and may annihilate with each other, accelerating the exciton dynamics decay. And the exciton‐exciton annihilation (EEA) could also lead to artificial enlargement of TAM image over time which would hinder the accurate measurement of exciton transport [29]. Therefore, exciton dynamics at different initial exciton density *n_0_* has been conducted as shown in Figure S19. It's found that the exciton dynamics is not changing even at an initial exciton density over 1 × 10^18^ cm^−3^, indicating that the excitons in SNH‐FB and SNEt‐FB crystals are not prone to annihilate. This also eliminates the interference from annihilation for the TAM imaging. This phenomenon is similar to our previous work on organic crystals based on other difluoroboron β‐diketonate derivatives [28]. The SNH‐FB and SNEt‐FB crystals also exhibit H‐type aggregation in specific crystallographic directions, which likely prevents measurable EEA under our experimental conditions. This property is in fact a notable advantage of the materials, as it allows high exciton mobility without significant annihilation losses.

The SNH‐FB and SNEt‐FB crystals were placed on substrates as shown in Figure 1 for the TAM imaging measurements. The exciton transport imaging of two crystals can be obtained by fixing the pump light and scanning the probe light relative to the pump light with a two‐dimensional scanning galvanometer. The TAM imaging measures the spatial distribution of the exciton population as a function of the pump‐probe delay time, based on that the transient absorption signal is proportional to the exciton density [28, 30, 31]. Because the pump and the probe lights can be approximated as Gaussian beams, the exciton population *n*(*x*,*y*,*t*) follows a two‐dimensional Gaussian distribution generated at the pump position (*x*_0_,*y*_0_). And the TAM image reflects the migration of excitons from the initial excitation position [31, 32]:

(1) $$\begin{array}{cc} & n\;\left(x,y,t\right)\\ & =N\cdot exp\left[-\frac{1}{2}{\left(\frac{x\cdot \cos\theta +y\cdot \sin\theta -x_{0}\cdot \cos\theta -y_{0}\cdot \sin\theta }{\sigma_{1}\left(t\right)}\right)}^{2}\right.\\ & \left.\;-\frac{1}{2}{\left(\frac{-x\cdot \sin\theta +y\cdot \cos\theta +x_{0}\cdot \sin\theta -y_{0}\cdot \cos\theta }{\sigma_{2}\left(t\right)}\right)}^{2}\right] \end{array}$$

*σ* is the variance of the exciton population distribution, and *θ* is the angle between the fast transport axis and the X‐axis of the image. Note that when *t* = 0 ps, *θ* = 0.

**FIGURE 4:**
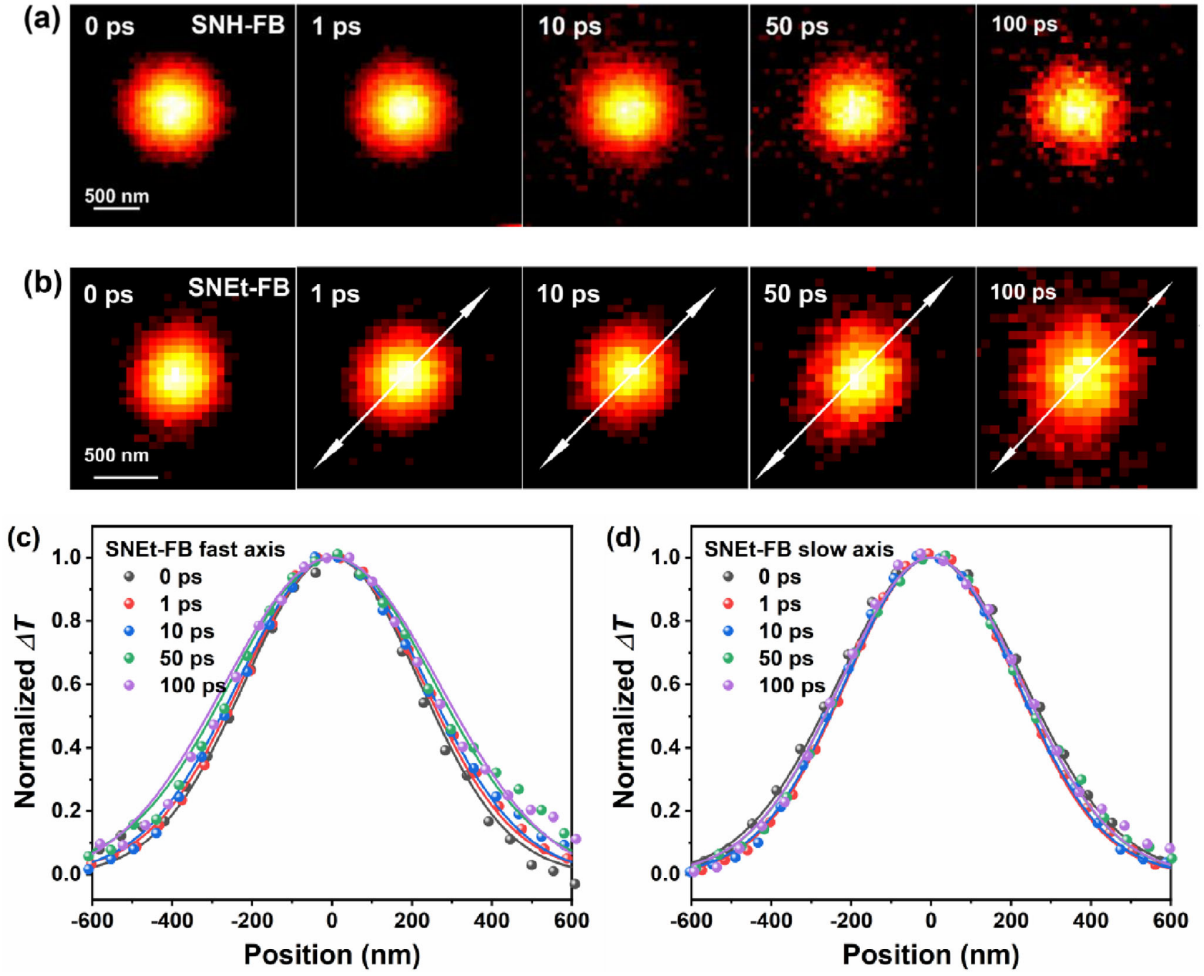
Exciton diffusion imaging for (a) SNH‐FB (*n_0_* = 2.1 × 10^18^ cm^−3^) and (b) SNEt‐FB (*n_0_* = 1.0 × 10^18^ cm^−3^) crystals obtained by TAM. The color scale represents the intensity of pump‐induced differential transmission (*ΔT*) of the probe, and every image has been normalized by the peak value. The white arrow in the TAM images indicates the fast transport axis. (c,d) Cross sections of the TAM images of SNEt‐FB crystal fitted with Gaussian functions along the white arrow in (b) and along the direction perpendicular to the arrow, respectively, with the maximum *ΔT* signal normalized.

**FIGURE 5:**
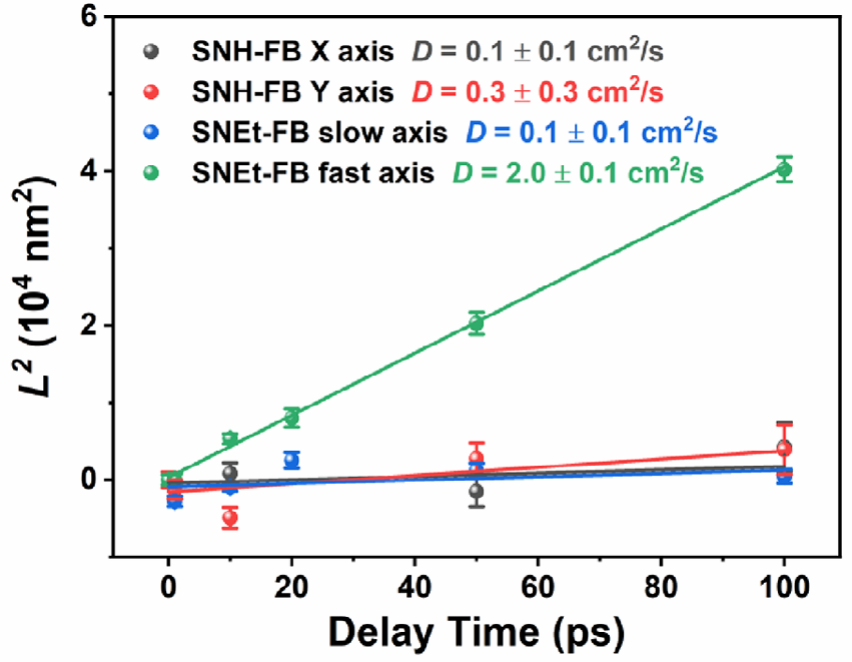
Time evolution and linear fitting of *L^2^* along the different axes for SNH‐FB and SNEt‐FB crystals. Error bars are the standard error estimated from the 2D Gaussian fitting to the spatial intensity distribution.

Figure 4a presents TAM exciton transport imaging of SNH‐FB crystal at different pump‐probe delay times. The image width remains relatively constant, indicating the absence of significant exciton diffusion. As shown in Figure S20, the Gaussian function was used to fit the cross sections of TAM images along the X‐axis and Y‐axis respectively, and the Gaussian width remains basically unchanged over time. By fitting the exciton transport imaging using Equation (1), the evolution of the exciton diffusion distance $L^{2}=\sigma_{\left(t\right)}^{2}-\;\sigma_{\left(0\right)}^{2}$ along the X and Y axes with delay time has been obtained, as shown in Figure 5. The calculated *L^2^* fluctuates around 0 with delay time, and the exciton diffusion constant *D* obtained by linear fitting (*L^2^* = 2*Dt*) is close to 0, which suggesting that the exciton transport rate in the SNH‐FB crystal is very slow. Note that the precision in determining the exciton diffusion distance is dictated by the smallest measurable change in the excited‐state population profile and not directly by the diffraction limit (not affected by the optical point spread function). This limit is ∼50 nm for the experimental conditions, as discussed in our previous reports [31, 33].

In contrast, as shown in Figure 4b, the exciton population distribution of the ethyl‐substituted SNEt‐FB crystal gradually becomes elliptical with time delay, indicating significant anisotropic exciton transport. The fast and slow axes of exciton transport have been determined by fitting with Equation (1). The fast axis is represented by the white arrow in Figure 4b, and the direction perpendicular to it represents the slow axis of exciton transport. Because the SNEt‐FB crystal was aligned in the TAM apparatus according to the PL image in Figure 1b, the fast transport axis shown in Figure 4b can match the a‐axis in Figure 1b (they have the same angle on the drawing plane). So it's found that the fast and slow axes correspond to the a and b axes of the crystal, respectively, as marked in Figure 1b. Figure 4c,d show the cross sections of the TAM images along the fast (a‐axis) and slow (b‐axis) axes of the SNEt‐FB crystal, respectively. It's evident that there is noticeable exciton diffusion along the fast axis but not along the slow axis. After linear fitting, the exciton diffusion constant of a‐axis is determined as 2.0 ± 0.1 cm^2^/s (Figure 5). However, the exciton diffusion constant of b‐axis obtained by fitting is 0.1 ± 0.1 cm^2^/s, which is almost 0. That means the exciton diffusion is strongly anisotropic and rapid along the a‐axis on the ab plane of the SNEt‐FB crystal. The lifetime (*τ*) of excitons for SNEt‐FB crystal is 261 ps which was obtained by fitting the TAM dynamics (Figure S21). The exciton diffusion length (*L*_D_) has been calculated using the equation $L_{D}=\sqrt{2D\tau }$. The resulting *L*_D_ value is 323 nm along the a‐axis of SNEt‐FB crystal.

To confirm the reliability of the TAM imaging measurement results, different crystals have been measured, and their PL microscopy images are shown in Figures S22 and S25. Figure S23 shows the TAM imaging data for the other SNH‐FB crystal, and it's similar to Figure 4a and Figure S20. The evolution of *L^2^* with time for both sets of data shows fluctuation around 0, as depicted in Figure S24. Furthermore, Figure S26 displays the TAM data of another SNEt‐FB crystal. The shape of image also becomes elliptical as the delay time increasing, indicating a notable anisotropy in exciton transport. The evolution of *L^2^* with time is illustrated in Figure S27. By performing linear fitting, we have determined that the exciton diffusion constant along the fast axis is approximately 1.9 cm^2^/s which is close to the first SNEt‐FB crystal. Conversely, the exciton diffusion along the slow axis direction is much slower than that of the fast axis.

The exciton diffusion constants *D* and anisotropy ratios *k* obtained in this work have been compared with representative prior studies (see Table S3). Note that anisotropy caused by special shapes (such as reducing material dimensions) is not within the scope of this comparison. It can be found that the exciton transport rate of SNEt‐FB is comparable with the maximum value of organic crystals, furthermore, the anisotropy of exciton transport is exceptional for molecular crystals. The fast axis diffusion constant is 2.0 ± 0.1 cm^2^/s, and the slow axis value is 0.1 ± 0.1 cm^2^/s. The resulting anisotropy ratio *k* is *D_fast_*/*D_slow_* = 20. However, due to the large relative uncertainty of the slow axis diffusivity (100% relative error), the uncertainty of the anisotropy ratio is highly asymmetric. The anisotropy ratio ranges approximately spanning from 9.5 to infinity. This analysis confirms that the exciton transport is strongly anisotropic, even though the complete suppression along the slow direction cannot be unambiguously established.

### 2.5 Theoretical Calculation of the Exciton Transport in SNH‐FB and SNEt‐FB Crystals

In order to consider the aggregation effect in the two crystals, we have performed calculations within the QM/MM framework. The excited‐state properties were studied by employing the density functional theory (DFT) and linear‐response time‐dependent DFT (LR‐TDDFT) methods. From the crystal structures, 3 × 3 × 3 clusters were constructed and used as the two‐layer QM/MM models (Figure S28), in which a central molecule was chosen as the QM region, described by the B3LYP functional [34] and the 6–31G* basis sets [35], while the surrounding molecules were included in the MM region, processed using the universal force field (UFF) [36]. The electrostatic interaction between the QM and MM regions was described by the electrostatic embedding model, and the atomic partial charges were generated by the restrained electrostatic potential (RESP) method [37].

Geometry optimizations and frequency analyses in the S_0_ and S_1_ states were first carried out. Based on the optimized S_0_ and S_1_ structures, the electronic excitation and emission energies were calculated at the same computational level. As shown in Table S4, the calculated electron excitation energy of SNH‐FB and SNEt‐FB are nearly identical. The emission energy of SNH‐FB exhibits a redshift of 27 nm relative to that of SNEt‐FB, which is smaller than the experimentally observed redshift (645 nm for SNH‐FB and 585 nm for SNEt‐FB). This discrepancy may be attributed to the presence of solvent molecules in the crystal structure of SNH‐FB, which could introduce defect states that are not adequately described by the computational methods employed. The difference between SNEt‐FB and SNH‐FB arises from the ethyl substitution, which changes the molecular packing and electronic structure, leading to a larger Stokes shift and narrower overlap in SNH‐FB.

Using the thermal vibration correlation function (TVCF) method [38], the absorption and emission spectra for SNEt‐FB were simulated (Figure S29). The obvious spectral overlap is conducive to the excited‐state energy transfer. In the incoherent regime, the excitation energy transfer (EET) rate *k_eet_* can be expressed by Fermi's golden rule:

(2) $$K_{eet}=\frac{2\pi }{\hbar }\;{\left|J_{DA}\right|}^{2}FCWD$$

where the ℏ is the reduced Planck constant. *J_DA_* is the excitonic coupling between the central molecule and the neighboring molecules, and FCWD denotes the Franck‐Condon weighted density of states. Within the Franck‐Condon formalism, the FCWD can be evaluated from the Franck‐Condon integrals of donor emission and acceptor absorption spectra. Therefore, the EET rate can be approximately written as [39, 40]:

(3) $$K_{eet}=\frac{2\pi }{\hbar }\;{\left|J_{DA}\right|}^{2}\int_{0}^{+\infty }d\omega \sigma_{emi}\left(\omega ,\;T\right)\sigma_{abs}\left(\omega ,\;T\right)$$

where *σ_emi_(ω, T)* and *σ_abs_(ω, T)* are the donor emission and acceptor absorption spectra, respectively, which are calculated using the TVCF formalism that accounts for molecular vibrations, reorganization energy, and electron‐phonon coupling [38, 41]. The spectral overlap integral $I=\int_{0}^{+\infty }d\omega \sigma_{emi}\left(\omega ,\;T\right)\sigma_{abs}\left(\omega ,\;T\right)$ can be evaluated by the MOMAP program [42]. For SNEt‐FB, the spectral overlap integral was directly calculated. In contrast, a direct calculation was avoided for SNH‐FB, as the presence of solvent molecules in its crystal structure may introduce defect states that are not reliably described by current computational methods. Instead, by fitting the area of the spectral overlap region between the absorption and emission bands in the experimental steady‐state spectra (Figure 2), the ratio of the spectral overlap integrals, *I_SNH‐FB, exp_*/*I_SNEt‐FB_*_, exp_, can be determined, thus allowing for the determination of the spectral overlap integral of Crystal SNH‐FB.

The cutoff value was set to 6 Å, meaning that exciton coupling between the central molecule and other surrounding molecules was calculated if the shortest interatomic distance between them was less than 6 Å. Here, we have calculated 26 coupling pairs for SNH‐FB and 27 coupling pairs for SNEt‐FB, respectively. The selected coupling pairs with the exciton couplings are shown in Figure S30, and the intermolecular centroid distances *R* and EET rates *k_eet_* with exciton couplings are listed in Tables S5 and S6. It should be noted that although the maximum coupling of SNH‐FB (167.6 meV) is greater than that of SNEt‐FB (148.9 meV) due to the shorter intermolecular centroid distance, the smaller spectral overlap (0.078 eV^−1^ for SNH‐FB and 0.478 eV^−1^ for SNEt‐FB) results in a relatively slower *k_eet_* for SNH‐FB (2.08 × 10^13^ s^−1^ for SNH‐FB and 1.01 × 10^14^ s^−1^ for SNEt‐FB). Correspondingly, when the exciton coupling values are comparable, the energy transfer rate between the central molecule and surrounding molecules in SNEt‐FB is at least five times higher than that in SNH‐FB. A relatively fast energy transfer rate leads to a large diffusion constant.

**FIGURE 6:**
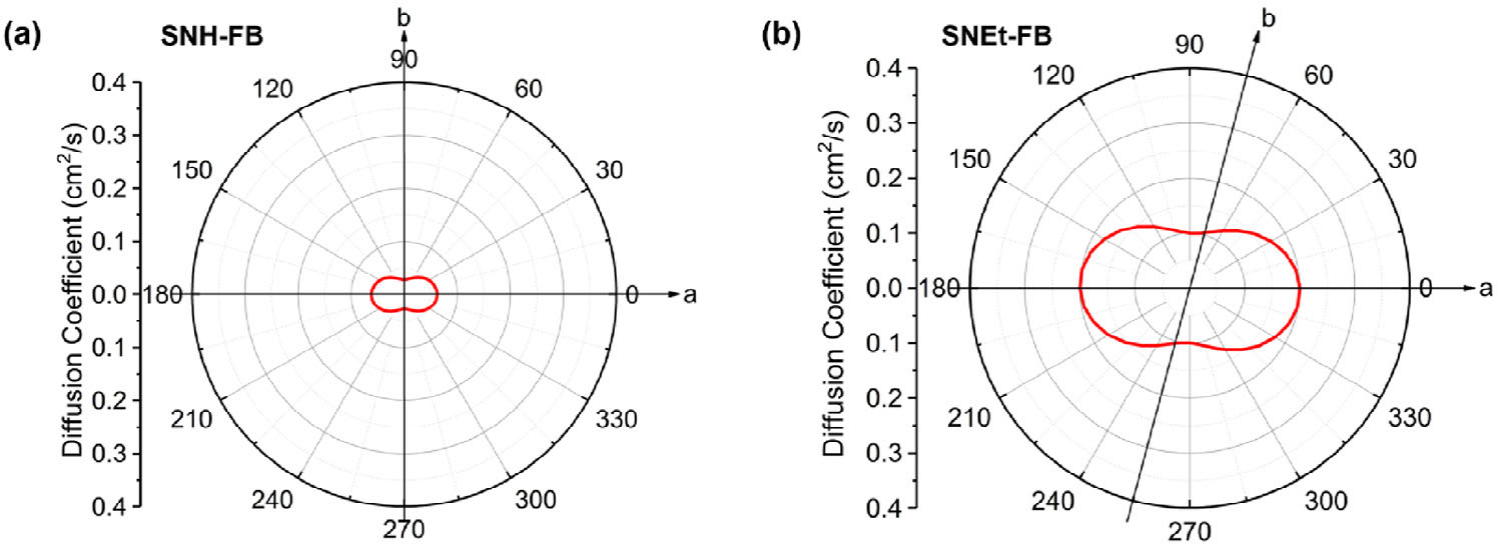
The calculated anisotropic diffusion constants in the ab plane of (a) SNH‐FB crystal and (b) SNEt‐FB crystal.

With the EET rates *k_eet_* in all the directions, we can simulate the exciton diffusion through the Monte Carlo method and calculate the exciton diffusion constants in two crystals. The calculated diffusion constants in the ab plane of SNH‐FB and SNEt‐FB crystals are shown in Figure 6. As expected, the diffusion constants along the lattice directions are obvious higher in the SNEt‐FB crystal than in the SNH‐FB crystal. This result is qualitatively consistent with the experimental observation that exciton diffusion occurs in the SNEt‐FB crystal, but not in the SNH‐FB crystal. Particularly, the SNEt‐FB crystal exhibits a maximum diffusion constant of 0.200 cm^2^/s along the a‐axis, while the minimum diffusion constant occurs at 90° (0.100 cm^2^/s).

It is worth noting that the calculated diffusion constant along the a‐axis (0.200 cm^2^/s) is approximately one order of magnitude smaller than the experimentally observed value (∼2.0 cm^2^/s), resulting in an underestimation of the diffusion anisotropy. This discrepancy is not unexpected, as our current calculations are based on the incoherent hopping regime and do not consider exciton delocalization—a key effect known to further enhance exciton transport rate by over one order of magnitude [28, 40]. Although the previous study was performed on a different crystal, the same exciton delocalization effect is expected to bridge the gap between experiment and theory for the strongly coupled diffusion direction in the present SNEt‐FB system. Therefore, our current calculations are intended for qualitative comparison—validating the observed anisotropy in SNEt‐FB and negligible diffusion in SNH‐FB—not for exact quantitative prediction.

The experimental and theoretical results presented above reveal a correlation between molecular structure and exciton transport behavior. In SNEt‐FB, the ethyl substitution induces an antiparallel and highly ordered molecular arrangement with reduced intermolecular distances along the a‐axis. This ordered packing favors large excitonic coupling between neighboring molecules. Consequently, the energy transfer rate along the a‐axis is significantly enhanced, leading to a diffusion constant of approximately 2.0 cm^2^/s. In contrast, SNH‐FB adopts a staggered packing mode with larger intermolecular separation and the crystal also contains solvent molecules that disrupt packing, introducing an additional variable beyond ethyl substitution. The spectral overlap integral is about six times smaller than that of SNEt‐FB, and the excitonic couplings are comparable but the overall calculated energy transfer rate is approximately five times slower. As a result, no significant exciton transport is observed in SNH‐FB within our experimental resolution. These findings demonstrate that introducing ethyl substituents to promote antiparallel ordering and to reduce intermolecular distances may be an effective molecular design strategy for achieving fast and anisotropic exciton transport in organic crystalline materials. Of course, the solvent molecules incorporated into the lattice may also have an impact on the exciton transport. The solvent molecules grow in the crystal because they have hydrogen bonds with NH. If the solvent molecules in the crystal are removed, the assembly structure of SNH‐FB itself will collapse and the crystal will weather. So it's impossible to evaluate the impact of solvent molecules on exciton diffusion while keeping the original molecular packing structure unchanged.

## 3 Conclusion

In summary, we have achieved strongly anisotropic and rapid exciton transport in thienopyrrole‐dicarbonyl fluoroboron organic crystals. Two organic crystals of SNH‐FB and SNEt‐FB have been synthesized to control the exciton transport rate and directions. The transient absorption imaging results indicate that no obvious exciton diffusion is observed in SNH‐FB crystals, but ethyl substituted SNEt‐FB crystals have significantly enhanced exciton diffusion constant (∼2 cm^2^/s) along only the a‐axis on the ab plane of the crystal. Theoretical calculations demonstrate that the coupling strength of molecules along different directions of the SNEt‐FB crystals is significantly different, making important contributions to the obvious anisotropy of exciton transport. This research contributes valuable insights to the development of organic semiconductor materials featuring not only rapid but also directed exciton transport.

## Conflicts of Interest

The authors declare no conflicts of interest.

## Supporting information




**Supporting File**: advs77660‐sup‐0001‐SuppMat.pdf.

## Data Availability

The data that support the findings of this study are available from the corresponding author upon reasonable request.
