# Supplementary material for: Strongly Anisotropic and Rapid Excitons Transport in Organic Crystal
Source: Adv Sci (Weinh). 2026 Sep 8:e77660. Online ahead of print. doi: 10.1002/advs.77660 (PMC13554491; doi:10.1002/advs.77660)
Supplement: Supplementary file 1 [file ADVS-9999-e77660-s001.pdf]

Supporting Information for

Strongly Anisotropic and Rapid Excitons Transport in Organic Crystal

Minjie Li,[1†] Zheng-Fei Liu,[1†] Jiajia Ma,[1†] Dayujia Huo,[1] Ganglong Cui,[1*] Qing-Zheng Yang,[1*] and Yan Wan[1*]

[1]College of Chemistry, Beijing Normal University, Beijing, 100875, P. R. China.

Corresponding author

*Yan Wan. E-mail: wanyan@bnu.edu.cn

*Qing-Zheng Yang. E-mail: qzyang@bnu.edu.cn

*Ganglong Cui. E-mail: ganglong.cui@bnu.edu.cn

[†]: Equal contribution

Table of Contents

1 Experimental Section 3
  1.1 Nuclear Magnetic Resonance 3
  1.2 Electrospray Ionization Mass Spectroscopy 3
  1.3 Single Crystal X-ray Diffraction 3
  1.4 Field Emission Transmission Electron Microscope (FE-TEM) 3
  1.5 Steady-state absorption and photoluminescence spectra measurements 4
  1.6 Femtosecond transient absorption spectra measurements 4
  1.7 Transient absorption microscopy measurements 5
  1.8 Theoretical Calculation 5
2 Synthesis and characterization of organic molecules 6
  2.1 Materials 6
  2.2 Synthesis 7
  2.3 NMR Spectra 10
  2.4 Mass Spectra 13
  2.5 X-ray Crystallography 14
  2.6 The selected area electron diffraction (SAED) of crystals 16
3 Spectra of organic crystals 17
  3.1 Figure S13- S16 TA spectra and dynamics 17
  3.2 Figure S17- S19 TAM dynamics 21
  3.3 Figure S20- S27 Exciton diffusion imaging and dynamics 22
  3.4 Table S3 Comparison of exciton transport anisotropy ratios 26
4 Theoretical calculation 27
  4.1 Figure S28 Setup of QM/MM Model 27
  4.2 Table S4 and Figure S29 Steady-state spectra 27
  4.3 Figure S30 and Table S5-S6 excitonic couplings 28
References 31

1. Experimental Section

Organic crystals SNH-FB and SNEt-FB were sealed in a nitrogen atmosphere by applying epoxy between two coverslips for optical measurements.

1.1 Nuclear Magnetic Resonance

Nuclear magnetic resonance (NMR) spectra were recorded on JEOL-400 or JEOL-600 spectrometer at room temperature. Chemical shift values (δ) and coupling constants (J) are reported in ppm and Hertz (Hz), respectively. The signals multiplicity for 1H NMR spectra are described using the following abbreviations: “s” (singlet), “d” (doublet), “t” (triplet), “dt” (doublet of triplets), “dd” (doublet of doublets) and “m” (multiplet).

1.2 Electrospray Ionization Mass Spectroscopy

High-resolution mass spectrometry experiments were performed on Bruker Solarix XR Fourier Transform Ion Cyclotron Resonance Mass Spectrometer.

1.3 Single Crystal X-ray Diffraction

Single crystal X-ray diffraction (SC-XRD) data were collected on a SuperNova Rigaku Oxford Diffraction diffractometer with Cu-Kα radiation, λ = 1.54184 Å. Using Olex2, the structures were solved with the SHELXTS5 structure solution program.

1.4 Field Emission Transmission Electron Microscope (FE-TEM)

TEM images and selected area electron diffraction (SAED) were obtained with a JEOL JEM-1011 transmission electron microscope. One drop of the as-prepared mixed solution was deposited on a carbon-coated copper grid and dried at room temperature.

1.5 Steady-state absorption and photoluminescence spectra measurements

The microzone absorption spectra were measured using a white light source (SLS201L/M, Thorlabs) and the light was focused on a single crystal through a 40× objective (NA = 0.6, Nikon). The transmitted light was collected through another objective (40×, NA = 0.65, Sunny Optical Technology (Group) Co., Ltd.) and then acquired by a spectrometer (USB2000+, Ocean Optics). The incident light spectrum was recorded through a blank coverslip and used as a reference to calculate the absorption spectrum.

The microzone photoluminescence spectra were measured using the output of a diode laser (453 nm, PDL 800-D, Picoquant) as excitation light, which was focused on a single crystal through a 40× objective (NA = 0.6, Nikon). The same objective was used to collect emission signal into a spectrometer (kymera 193i, Andor) and an EMCCD camera (Newton EMCCD, Andor). A 500 nm long pass filter was used to filter out stray excitation light.

1.6 Femtosecond transient absorption spectra measurements

Our microzone femtosecond transient absorption device is based on the commercial ultrafast transient absorption spectrometer (Harpia-TA, Light Conversion). An 800 nm output produced by an amplified femtosecond Ti: sapphire laser (40 fs, 1kHz, Coherent Astrella) was divided into two beams. The pump light with a wavelength of 400 nm was generated by an optical parametric amplifier (TOPAS-C, Coherent), and the other beam was focused on a sapphire crystal to produce supercontinuous white light as the probe light. The pump and probe light were collinear in space through a beam splitter and then focused on a single crystal by a reflective objective (LMM-25X, Thorlabs). The single crystal was located by a microscope translation stage and a CMOS camera (EO-5012, Edmund). The transmitted light was

collected by the spectrometer in Harpia-TA.

1.7 Transient absorption microscopy measurements

Exciton transport imaging in organic single crystals was measured using a homemade transient absorption microscopy setup, which has been reported earlier[1]. The output of a amplified femtosecond laser (1030 nm, 600 kHz, PHAROS, Light Conversion) was imported into a two-channel optical parametric amplifier (ORPHEUS-Twins, Light Conversion). Then the output from one channel was used as the pump light (400 nm) and the other one as the probe light (820 nm). The pump light was modulated by an acousto-optic modulator (Gooch and Housego). The delay time of the probe light was changed by a translation stage (DDS600-E, Thorlabs), and the position of the probe light spot locating on the sample was changed by a two-dimensional galvanometer mirror (GVS012, Thorlabs). The pump and probe light were collinearly focused on the sample through a 60× objective (CFI Apo TIPF, NA = 1.49, Nikon). The transmitted probe light was acquired by an avalanche photodiode (Hamamatsu). The pump light induced change in the transmission intensity of the probe light (ΔT) was extracted by a lock-in amplifier. The focused pump spot size was approximately 209 nm, obtained by scanning beam across a Ag nanowire (width = 90 nm) and measuring the transmission intensity. Initial exciton density n_0 was calculated using the equation of $n_0 = \frac{F\alpha}{h\omega}$, in which F is the pump fluence (pump energy / pump facula area), $h\omega$ is the photon energy. The absorption coefficient $\alpha = \frac{A}{0.434d}$. d is the thickness of sample and A is the absorbance[2].

1.8 Theoretical Calculation

The excitonic coupling between the molecules in the crystal and the central molecule is calculated at the B3LYP/ 6-31G* level using the direct coupling (DC) method[3,4] in the QChem program[5]. With spectral

overlap integral and exciton couplings, the excitation energy transfer rate k_{eet} from the central molecule to another one can be obtained according to the Fermi's golden rule. Subsequently, we simulated the exciton diffusion through the Monte Carlo method. We choose a molecule as the initial exciton center denoted as m, and the exciton hops from m to another molecule n at rate k_{eet}^{n} with a hopping probability $P^{i} = \frac{k_{eet}^{n}}{\sum_{i=1}^{N} k_{eet}^{n}}$, where N is the total number of energy- accepting molecules. The hopping distance is intermolecular centroid distances. Before each hopping, a random number r is generated from a uniform distribution between 0 and 1, and if the random number falls within the range of $\sum_{n=1}^{j-1} P_{mn} \le r \le \sum_{n=1}^{j} P_{mn}$, the exciton jumps in the j direction. After each hopping, the simulation time is increased by $\frac{1}{\sum_{n} k_{mn}}$, and the simulation will be stopped when the total simulation time is attained[6]. Here, we set the simulation time as 1000 ns and obtain 2000 exciton diffusion trajectories. Then, the diffusion constant along the lattice directions in the crystal can be obtained as $D = \frac{\langle l^2(t) \rangle}{2t}$, where $\langle l^2(t) \rangle$ is the mean-square displacement.

2. Synthesis and characterization of molecules and crystals

2.1 Materials

General reagents and solvents were purchased from commercial suppliers and used without any purification, unless otherwise noted. 4H-thieno[3,2-b]pyrrole-5-carboxylate, di-tert-butyl dicarbonate, 4-methoxyacetophenone, 4-dimethylaminopyridine, iodoethane, boron trifluoride diethyl etherate, triethylamine, potassium hydroxide and sodium hydride were purchased from Sun Chemical Technology (Shanghai) Co. Ltd. All solvents were purchased from Beijing Chemical Reagent Plant.

2.2 Synthesis

Synthesis of 4-(4H-thieno[3,2-b]pyrrole-5-yl)-6-(4-methoxyphenyl)-2,2-difluoro-2H-1,3,2-dioxaborinin-1-ium-2-uide (SNH-FB)

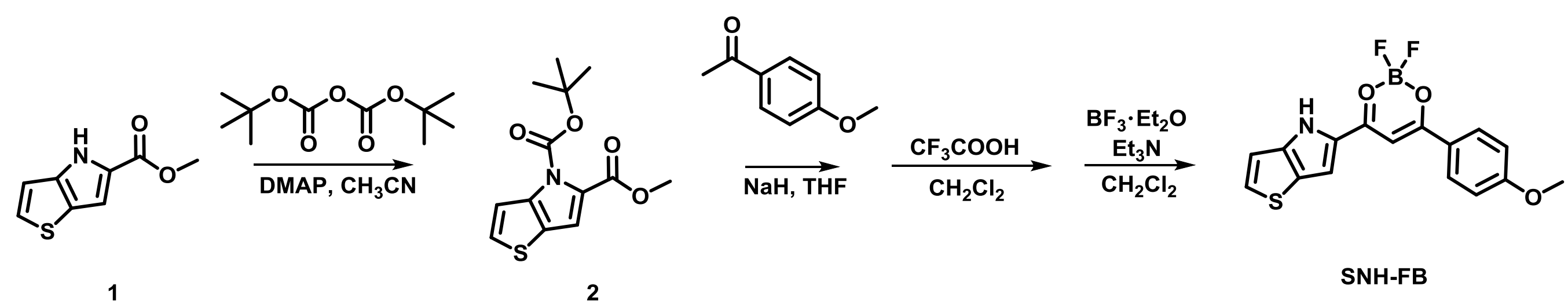

Figure S1. Synthetic route of SNH-FB.

Synthesis of 4-(tert-butyl) 5-methyl 4H-thieno[3,2-b]pyrrole-4,5-dicarboxylate (compound 2): In a vacuum dried 100 mL round-bottomed flask, Boc_2O (1.3 g, 6.1 mmol, 1.1 eq) and DMAP (0.13 g, 1.1 mmol, 0.20 eq) were added to a well-stirred solution of methyl 4H-thieno[3,2-b]pyrrole-5-carboxylate (compound **1**) (1.0 g, 5.5 mmol, 1.0 eq) in acetonitrile (30 mL) at 25 °C under argon atmosphere. After being stirred overnight, the reaction mixture was concentrated under reduced pressure. The obtained crude material was purified by column chromatography (petroleum ether/ethyl acetate = 10:1) to afford the product in 90% yield (1.4 g, 5.0 mmol) as a colorless oil. ^{1}H NMR (600 MHz, $CDCl_3$, 298 K) δ 7.37 (d, J = 5.3 Hz, 1H), 7.30 (d, J = 5.3 Hz, 1H), 7.10 (s, 1H), 3.87 (s, 3H), 1.61 (s, 9H).

Synthesis of SNH-FB:

Step 1: 4-Methoxyacetophenone (187.0 mg, 1.2 mmol) and THF (5.0 mL) were added to a 25.0 mL round-bottom flask and purged with nitrogen for 10 mins. Then NaH (57-63% oil dispersion, 188.0 mg, 4.7 mmol) was added to the resulting solution, and stirred under nitrogen at 60 °C for 30 min, followed by slow addition of compound **2** (350.0 mg, 1.2 mmol). The resulting mixture was refluxed under nitrogen

for 20 hours. The reaction mixture was allowed to cool to room temperature before being quenched carefully with water (10.0 mL) in ice bath. The pH was adjusted to 3 with HCl (aq). The aqueous suspension was extracted with ethyl acetate (3× 30.0 mL). The organic phase was dried over anhydrous sodium sulfate, and concentrated under reduced pressure to obtain the crude product as a brown oil. The crude product was directly used for next step without further purification.

Step 2: To a solution of the crude product in 10.0 mL dichloromethane at room temperature was added slowly 3.0 mL TFA. After being stirred for 2 h, the reaction mixture was concentrated under reduced pressure. The crude product was directly used for next step without further purification.

Step 3: To a solution of the crude product in 10.0 mL dichloromethane was added 1.0 mL Et_3N. After stirring for 10 min at room temperature, 1.0 mL $BF_3 \cdot Et_2O$ was added. The solution was stirred for another 2 hours in the dark. Water was added to the solution, and the organic layers was collected, washed with water, dried over anhydrous sodium sulfate, and concentrated under reduced pressure. The crude product was purified by column chromatography (petroleum ether/dichloromethane = 1:3) to yield SNH-FB (42.0 mg, 10%) as a yellow solid. ^1H NMR (600 MHz, DMSO-d_6, 298K) δ 8.20 (d, J = 8.7 Hz, 2H), 8.01 (s, 1H), 7.85 (d, J = 4.9 Hz, 1H), 7.53 (s, 1H), 7.17 (d, J = 8.4 Hz, 2H), 7.11 – 7.08 (m, 1H), 3.90 (s, 3H). ^{13}C NMR (151 MHz, DMSO-d_6, 298K) δ 176.00, 171.51, 164.43, 147.70, 136.02, 130.58, 126.52, 124.20, 114.76, 113.08, 111.92, 91.73, 55.84. HR-MS (ESI) $C_{16}H_{11}BF_2NO_3S^-$ $[M-H]^-$, Calculated: 346.0599; Found: 346.0528.

Synthesis of 4-(4-ethyl -thieno[3,2-b]pyrrole-5-yl)-6-(4-methoxyphenyl)-2,2-difluoro-2H-1,3,2-dioxaborinin-1-ium-2-uide (SNEt-FB)

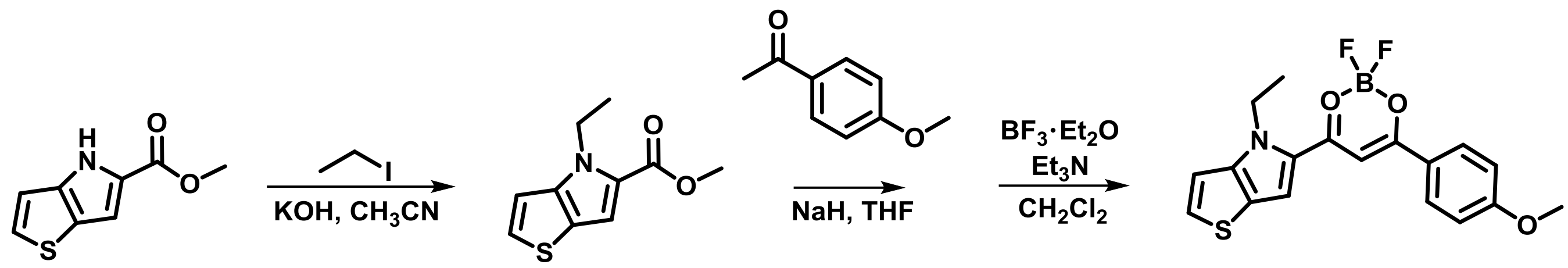

Figure S2. Synthetic route of SNEt-FB.

Synthesis of methyl 4-ethyl-4H-thieno[3,2-b]pyrrole-5-carboxylate (compound 3): To a solution of compound **1** (1.0 g, 5.5 mmol) in 50.0 mL acetonitrile was added potassium hydroxide (924.0 mg, 16.5 mmol) and iodoethane (1.3 g, 8.2 mmol). The reaction mixture was stirred at room temperature for 5 hours. The resulting mixture was extracted with dichloromethane (3× 50.0 mL) and the organic phase was collected, dried over anhydrous sodium sulfate and concentrated in vacuum. The crude product was purified by column chromatography (petroleum ether/dichloromethane = 3:1) to yield compound **3** (1.0 g, 90%) as a light-yellow oil. ^{1}H NMR (400 MHz, $CDCl_3$, 298 K) δ 7.33 (d, J = 5.4 Hz, 1H), 7.17 (s, 1H), 6.94 (d, J = 5.4 Hz, 1H), 4.54 (q, J = 7.1 Hz, 2H), 3.86 (s, 3H), 1.41 (t, J = 7.2 Hz, 3H).

Synthesis of SNEt-FB: The compound was synthesized according to the similar procedure described above in synthesis of compound SNH-FB by using compound **3** and 4-methoxyacetophenone to afford SNEt-FB (yield: 50%) as a yellow solid. ^{1}H NMR (400 MHz, $CDCl_3$, 298 K) δ 8.04 (d, J = 9.0 Hz, 2H), 7.54 (d, J = 5.4 Hz, 1H), 7.46 (d, J = 0.7 Hz, 1H), 7.07 – 6.93 (m, 3H), 6.86 (s, 1H), 4.61 (q, J = 7.2 Hz, 2H), 3.91 (s, 3H), 1.51 (t, J = 7.1 Hz, 3H). ^{13}C NMR (101 MHz, DMSO-d_6, 298 K) δ 176.05, 173.26, 164.85, 151.05, 136.88, 131.10, 129.54, 124.91, 124.84, 116.33, 115.20, 111.90, 93.25, 56.34, 44.36, 16.61. HR-MS (ESI) $C_{18}H_{16}BF_2NO_3S$, Calculated: 375.0912; Found: 375.0921.

2.3. NMR Spectra

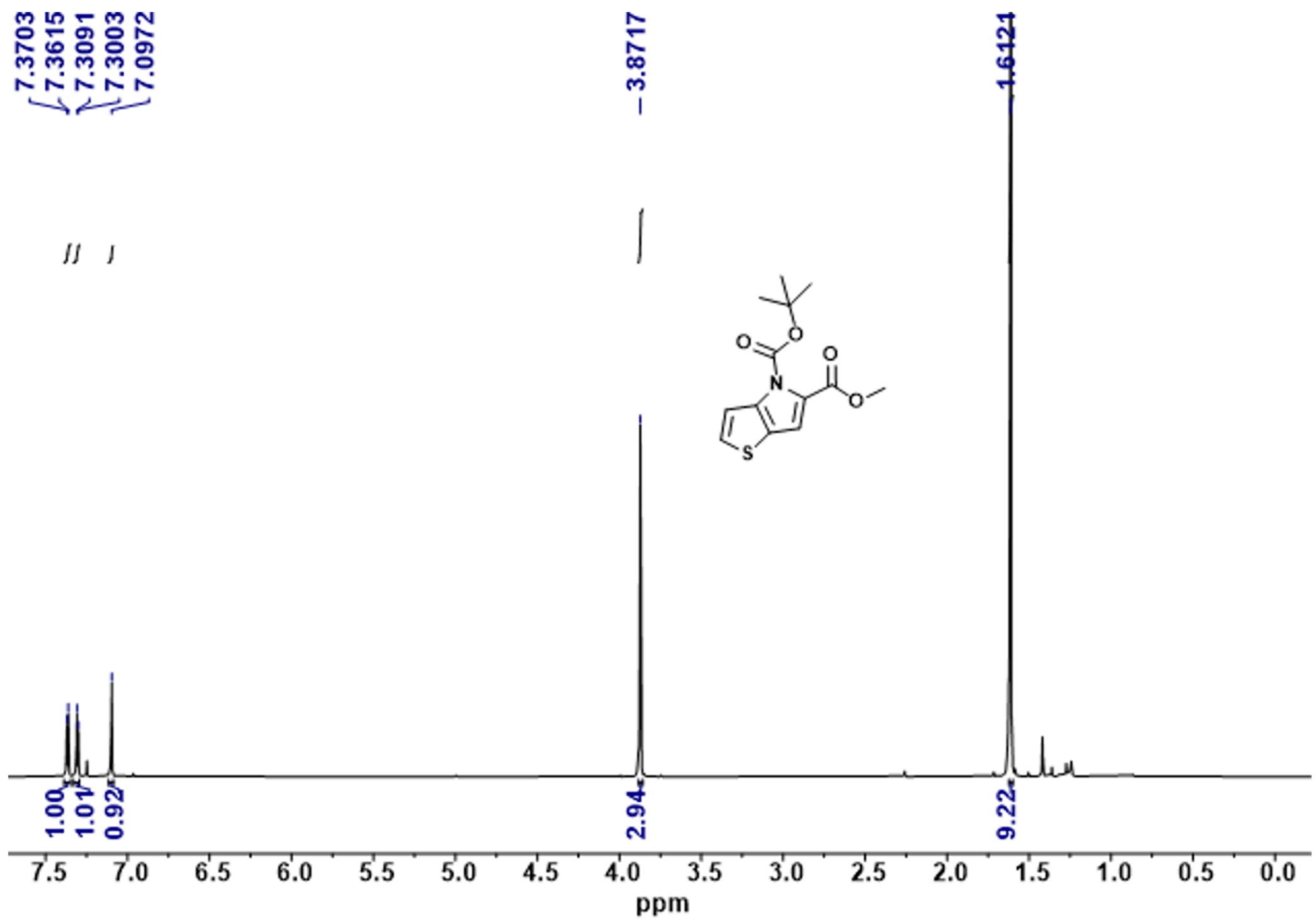

Figure S3. The ^{1}H NMR spectrum of compound 2 in $CDCl_3$ (600 MHz, 298 K).

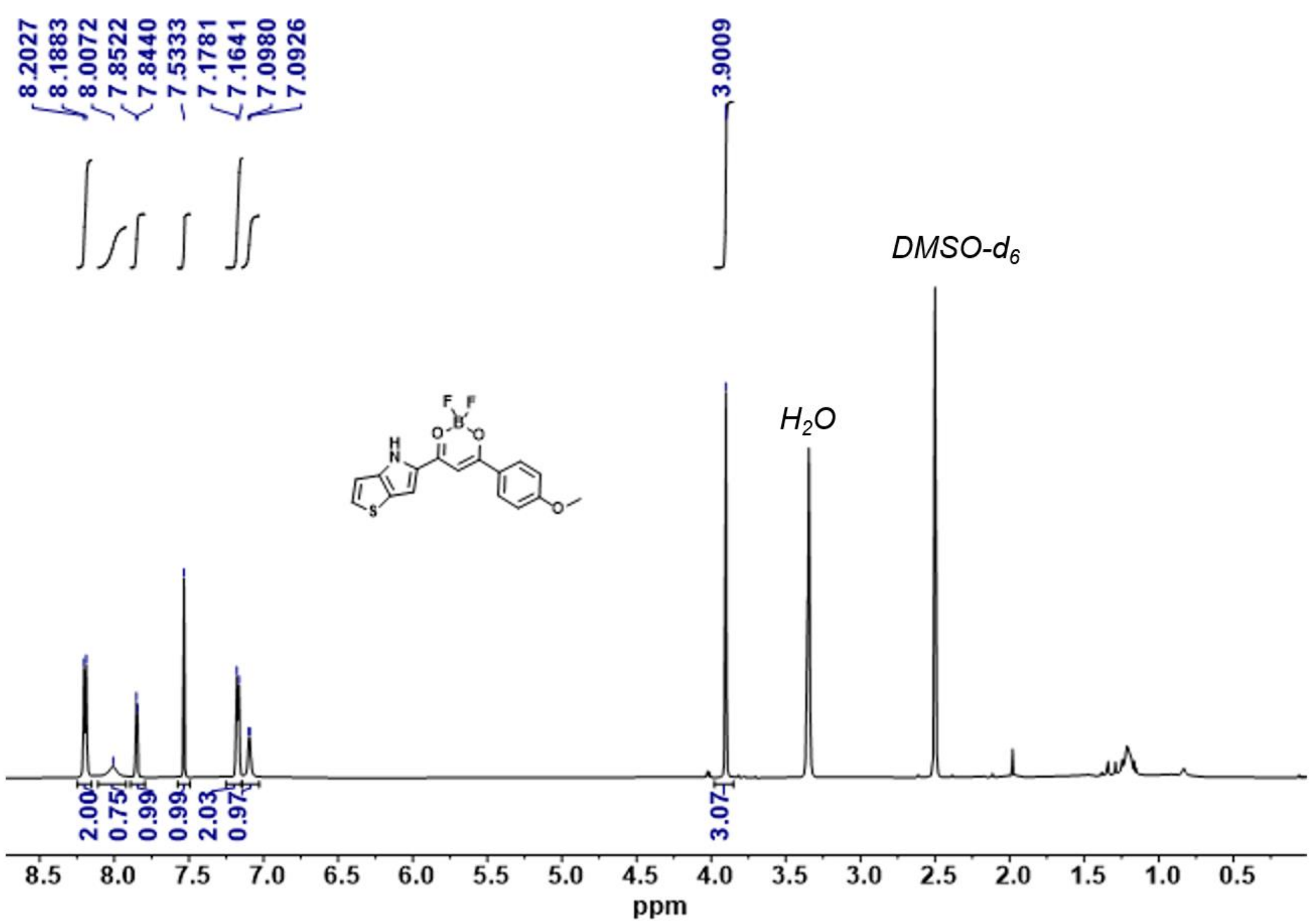

Figure S4. The ^{1}H NMR spectrum of compound SNH-FB in DMSO-d_6 (600 MHz, 298 K).

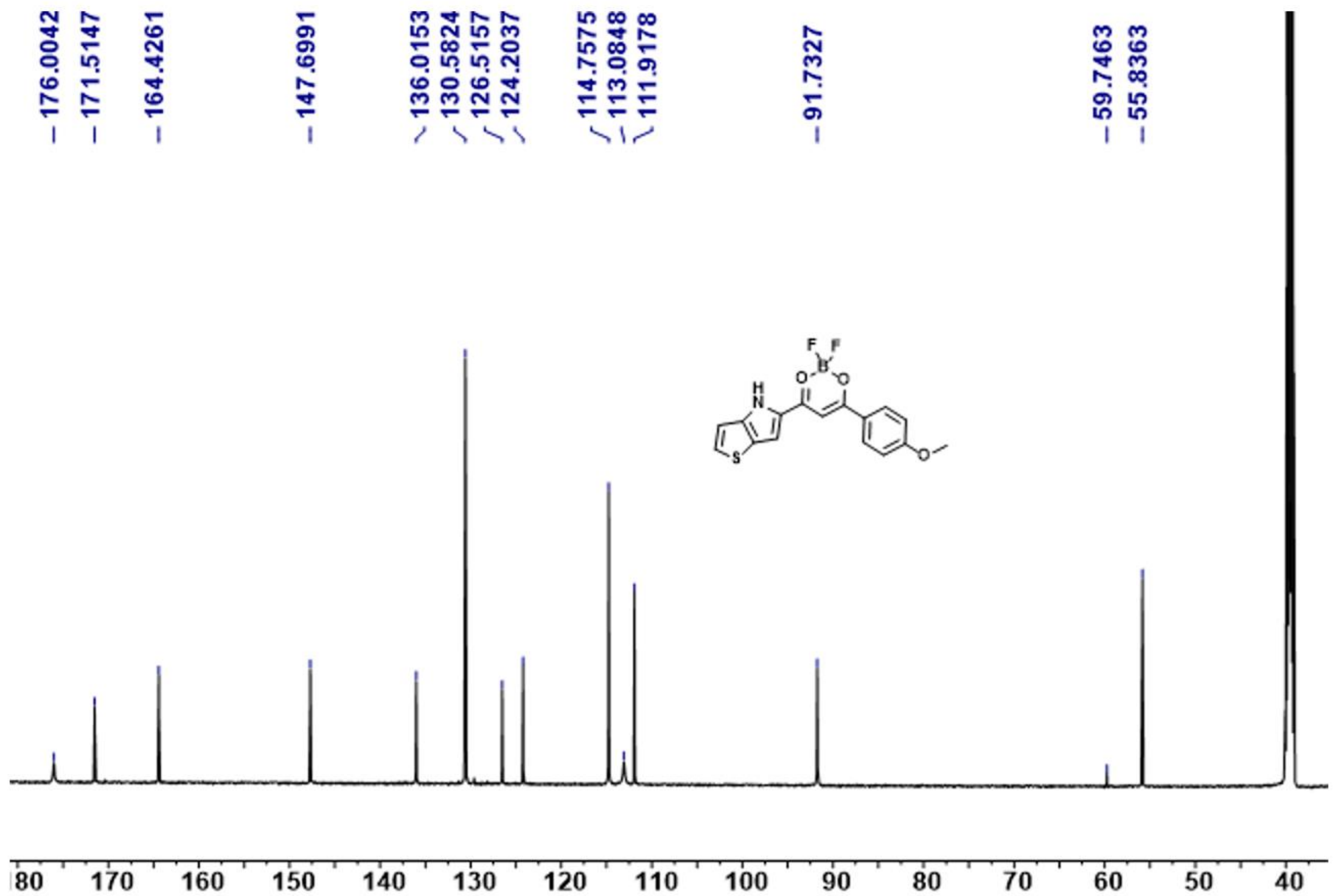

Figure S5. The ^{13}C NMR spectrum of SNH-FB in DMSO-d_6 (101 MHz, 298 K).

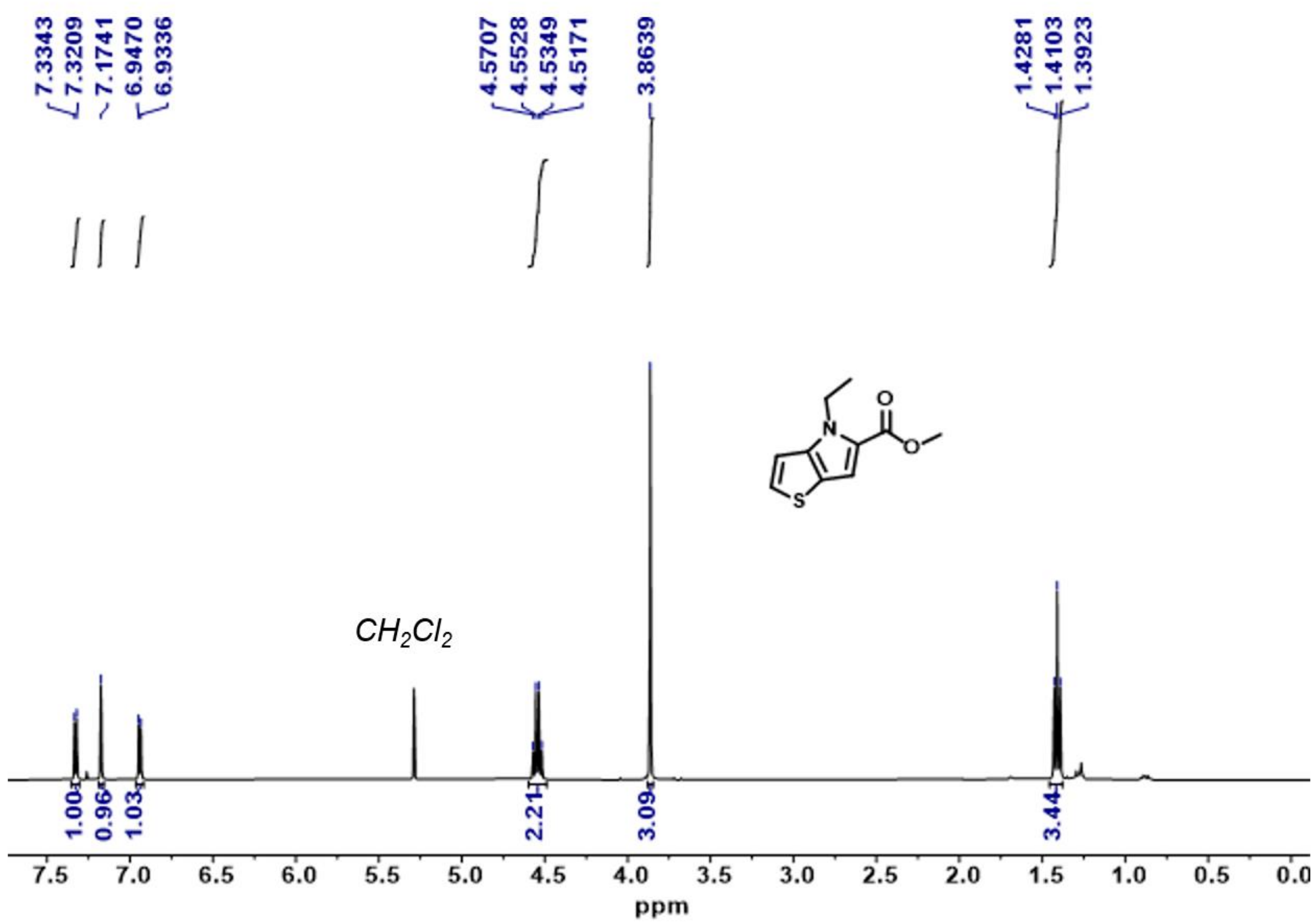

Figure S6. The ^1H NMR spectrum of compound 3 in $CDCl_3$ (400 MHz, 298 K).

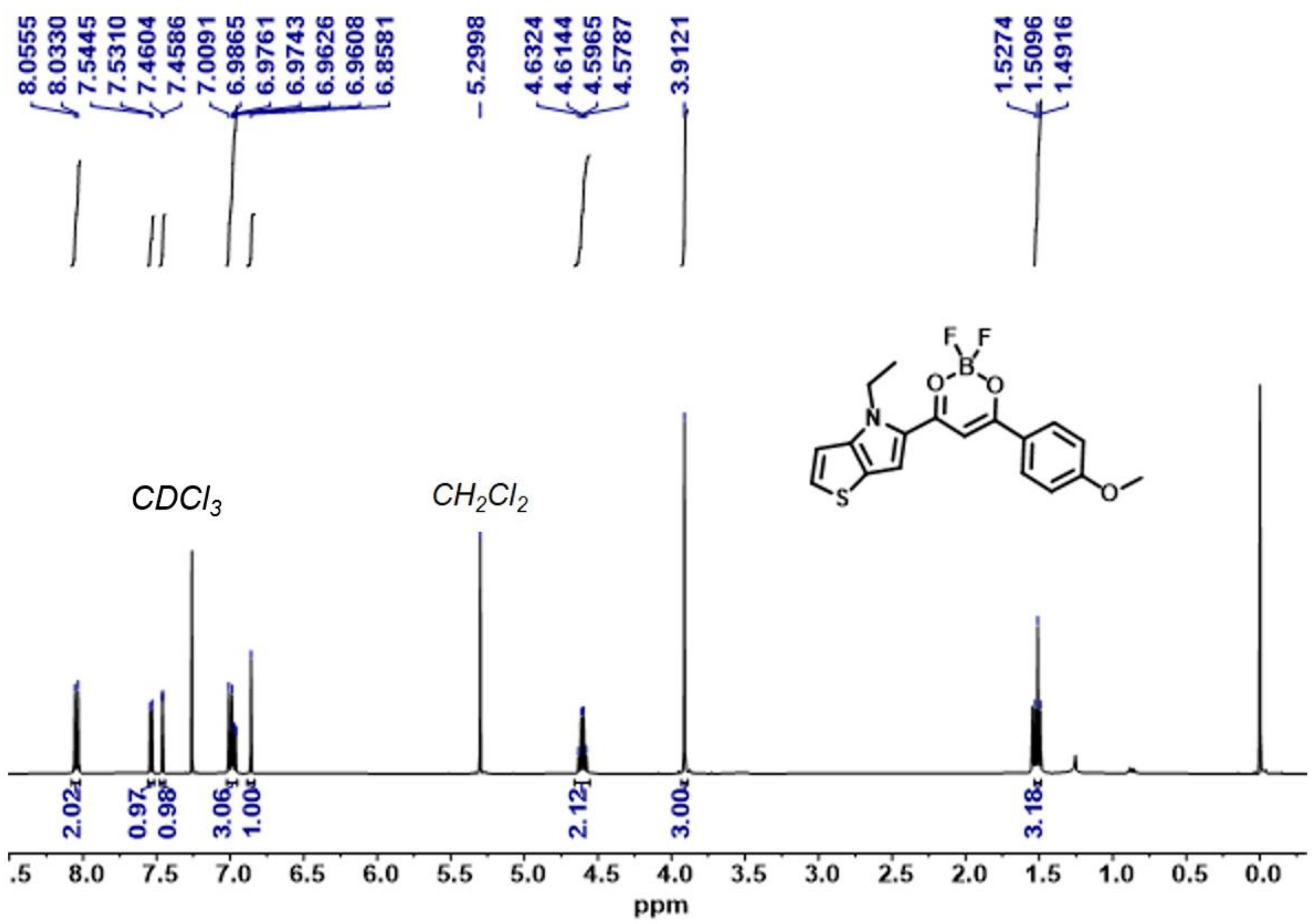

Figure S7. The ^{1}H NMR spectrum of compound SNEt-FB in $CDCl_3$ (400 MHz, 298 K).

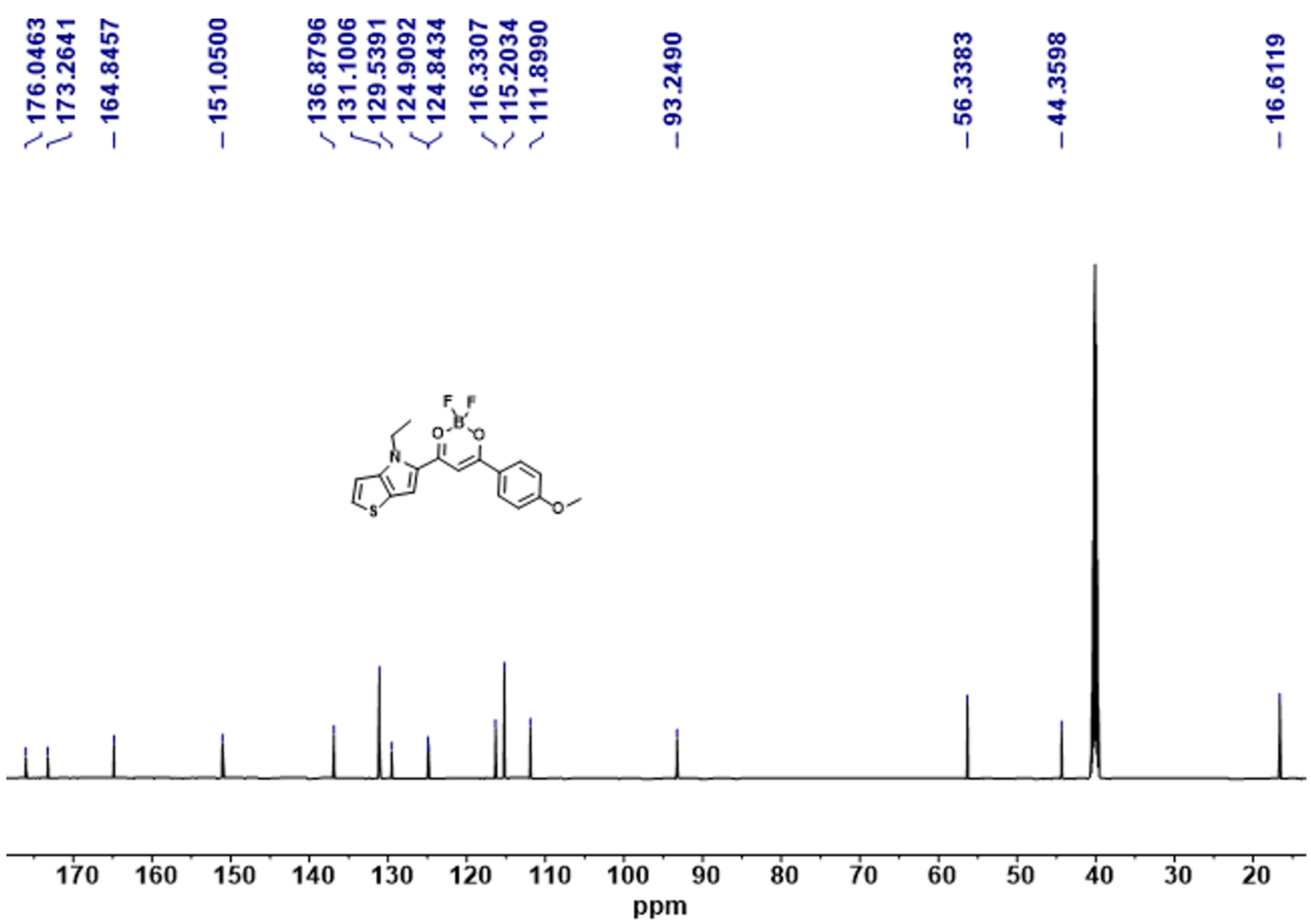

Figure S8. The ^{13}C NMR spectrum of SNEt-FB in DMSO-d_6 (101 MHz, 298 K).

2.4. Mass Spectra

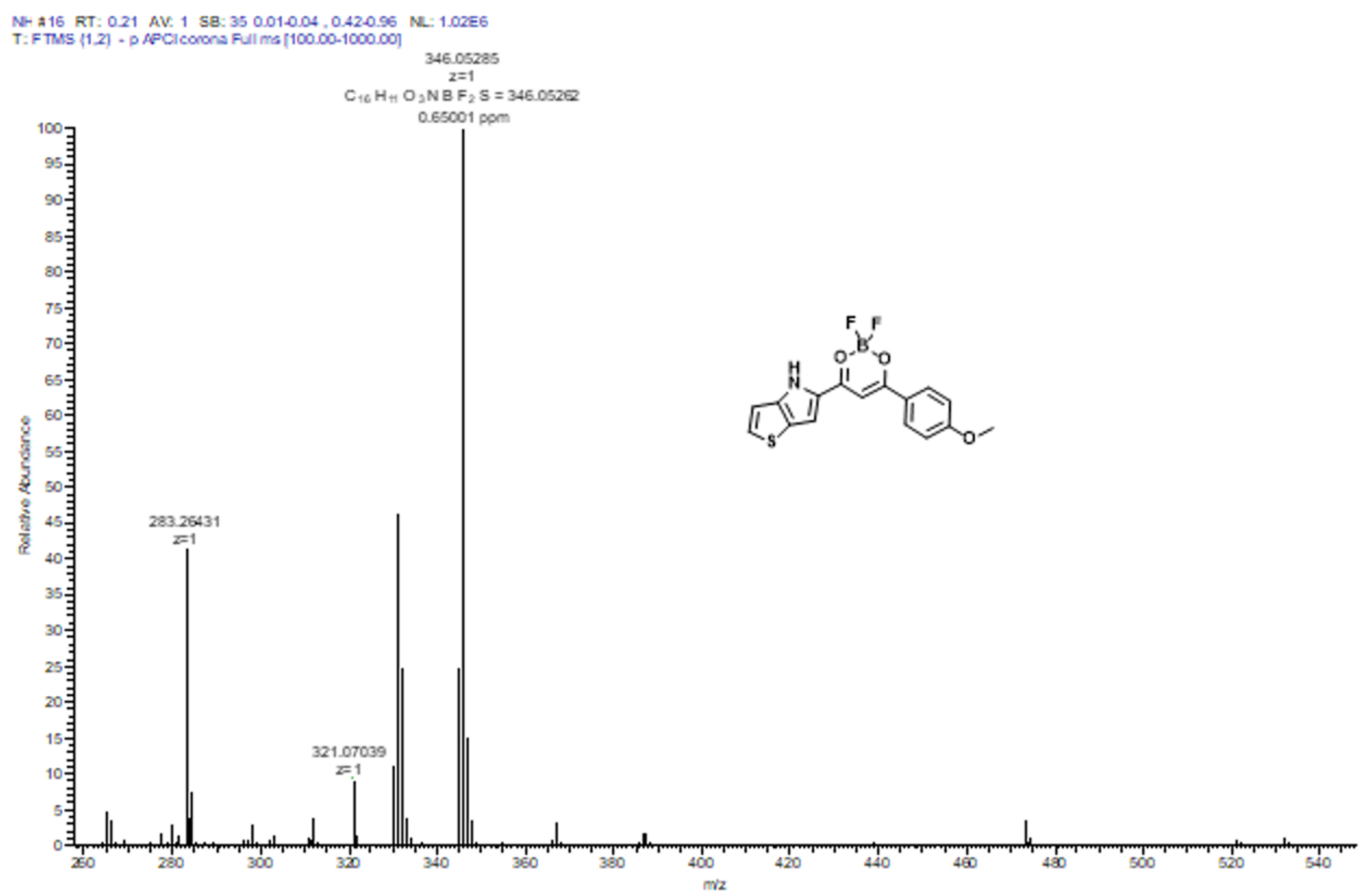

Figure S9. Experimental ESI-MS spectra of SNH-FB ([M-H]$^-$, Calculated: 347.0599).

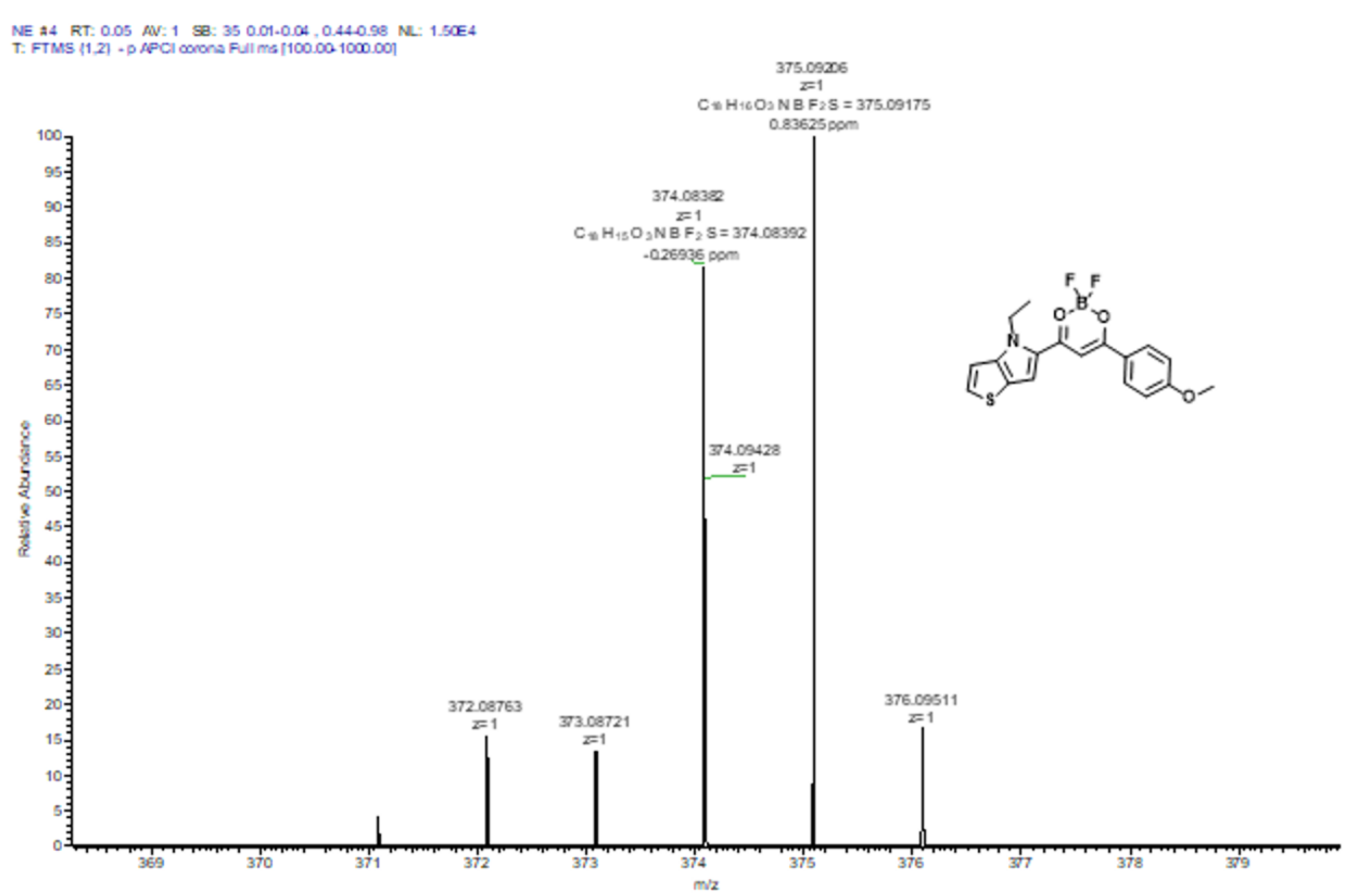

Figure S10. Experimental ESI-MS spectra of SNEt-FB ([M-H]$^-$, Calculated: 375.0912).

2.5. X-ray Crystallography

Single crystals of SNH-FB and SNEt-FB suitable for X-ray crystallography were obtained by slow evaporation of their solution in mixed solvents of dichloromethane and *n*-hexane (v/v = 2:1). CCDC deposition numbers of SNH-FB and SNEt-FB are 2413281 and 2413280, respectively.

Table S1. Crystal data and structure refinement for SNH-FB**.**

Identification code	**SNH-FB**
Empirical formula	$C_{17}H_{14}BCl_2F_2NO_3S$
Formula weight	432.06
Temperature	100.00 K
Wavelength	1.54184 Å
Crystal system, space group	Orthorhombic, *P*bca
Unit cell dimensions	a = 21.1573(2) Å, α = 90°
	b = 7.98740(8) Å, β = 90°
	c = 21.2600(2) Å, γ = 90°
Volume	3592.77(7) Å^3
Z, Calculated density	8, 1.598 g/cm^3
Absorption coefficient	4.701 mm^{-1}
F (000)	1760.0
Crystal size	0.2 × 0.08 × 0.05 mm^3
2θ range for data collection	8.318 to 153.136°
Index ranges	$-25 \leqslant h \leqslant 26, -10 \leqslant k \leqslant 7, -26 \leqslant l \leqslant 26$
Reflections collected	26263
Independent reflections	3690 [R_{int} = 0.0626, R_{sigma} = 0.0327]
Data/restraints/parameters	3690/0/245
Goodness-of-fit on F^2	1.225
Final R indexes [I>=2σ (I)]	R_1 = 0.0399, wR_2 = 0.1396
Final R indexes [all data]	R_1 = 0.0428, wR_2 = 0.1429
Largest diff. peak/hole / e	0.43/-0.40 Å^{-3}

Table S2. Crystal data and structure refinement for SNEt-FB.

Identification code	**SNEt-FB**
Empirical formula	$C_{54}H_{48}B_3F_6N_3O_9S_3$
Formula weight	1125.56
Temperature	100.00 K
Wavelength	1.54184 Å
Crystal system, space group	Triclinic, *P*-1
Unit cell dimensions	a = 8.94545 (10) Å, α = 83.5041(9)°
	b = 8.98505(12) Å, β = 89.0320(9)°
	c = 32.5232(3) Å, γ = 75.6694(11)°
Volume	2516.30(5) $Å^3$
Z, Calculated density	2, 1.486 g/cm^3
Absorption coefficient	2.080 mm^{-1}
F (000)	1164.0
Crystal size	0.9 × 0.7 × 0.5 mm^3
2θ range for data collection	8.208 to 153.006°
Index ranges	-10 ⩽ h ⩽ 11, -11 ⩽ k ⩽ 10, -40 ⩽ l ⩽ 41
Reflections collected	33053
Independent reflections	10162 [R_{int} = 0.0462, R_{sigma} = 0.0429]
Data/restraints/parameters	10162/0/710
Goodness-of-fit on F^2	1.083
Final R indexes [I>=2σ (I)]	R_1 = 0.0411, wR_2 = 0.1104
Final R indexes [all data]	R_1 = 0.0493, wR_2 = 0.1136
Largest diff. peak/hole / e	0.36/-0.48 $Å^{-3}$

2.6. The selected area electron diffraction (SAED) of crystals

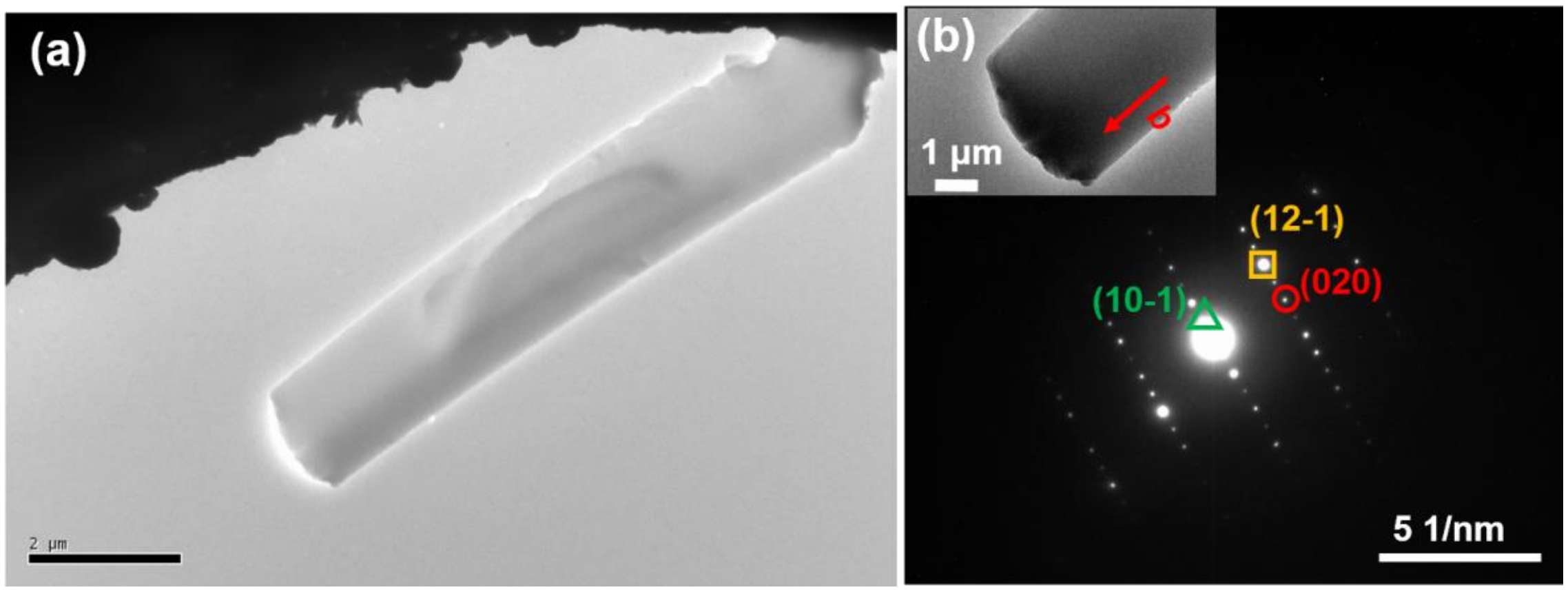

Figure S11. (a) TEM image and (b) corresponding SAED pattern of an SNH-FB crystal. The diffraction spots marked by the green triangle, yellow square, and red circle are indexed as the (10-1), (12-1), and (020) reflections, with corresponding *d*-spacings of 15.2, 3.74, and 3.96 Å, respectively. In the SAED pattern, the angles between the reciprocal-lattice vectors from the center to the (10-1) and (12-1) reflections and from the center to the (12−1) and (020) reflections are 71.3° and 19.7°, respectively. The crystallographic *b*-axis is indicated by the red arrow in the inset of (b).

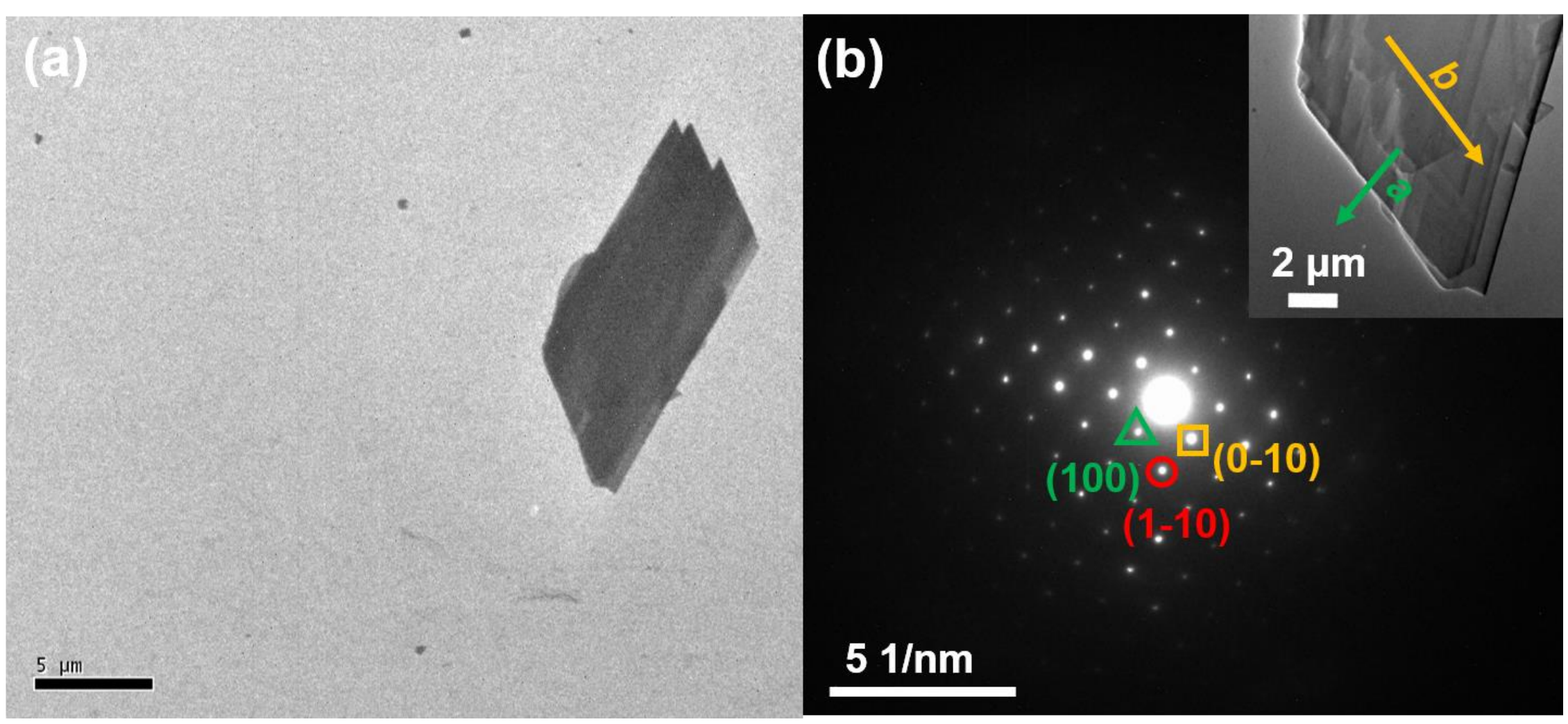

Figure S12. (a) TEM image and (b) corresponding SAED pattern of an SNEt-FB crystal. The diffraction spots marked by the green triangle, red circle, and yellow square are indexed as the (100), (1-10), and (0-10) reflections, with corresponding *d*-spacings of 8.71, 5.28, and 8.37 Å, respectively. In the SAED pattern, the angles between the reciprocal-lattice vectors from the center to the (100) and (1-10) reflections and from the center to the (1-10) and (0-10) reflections are 38.75° and 35.83°, respectively. The crystallographic *a*-axis and *b*-axis are indicated by the green arrow and yellow arrow in the inset of (b).

3. Spectra of organic crystals

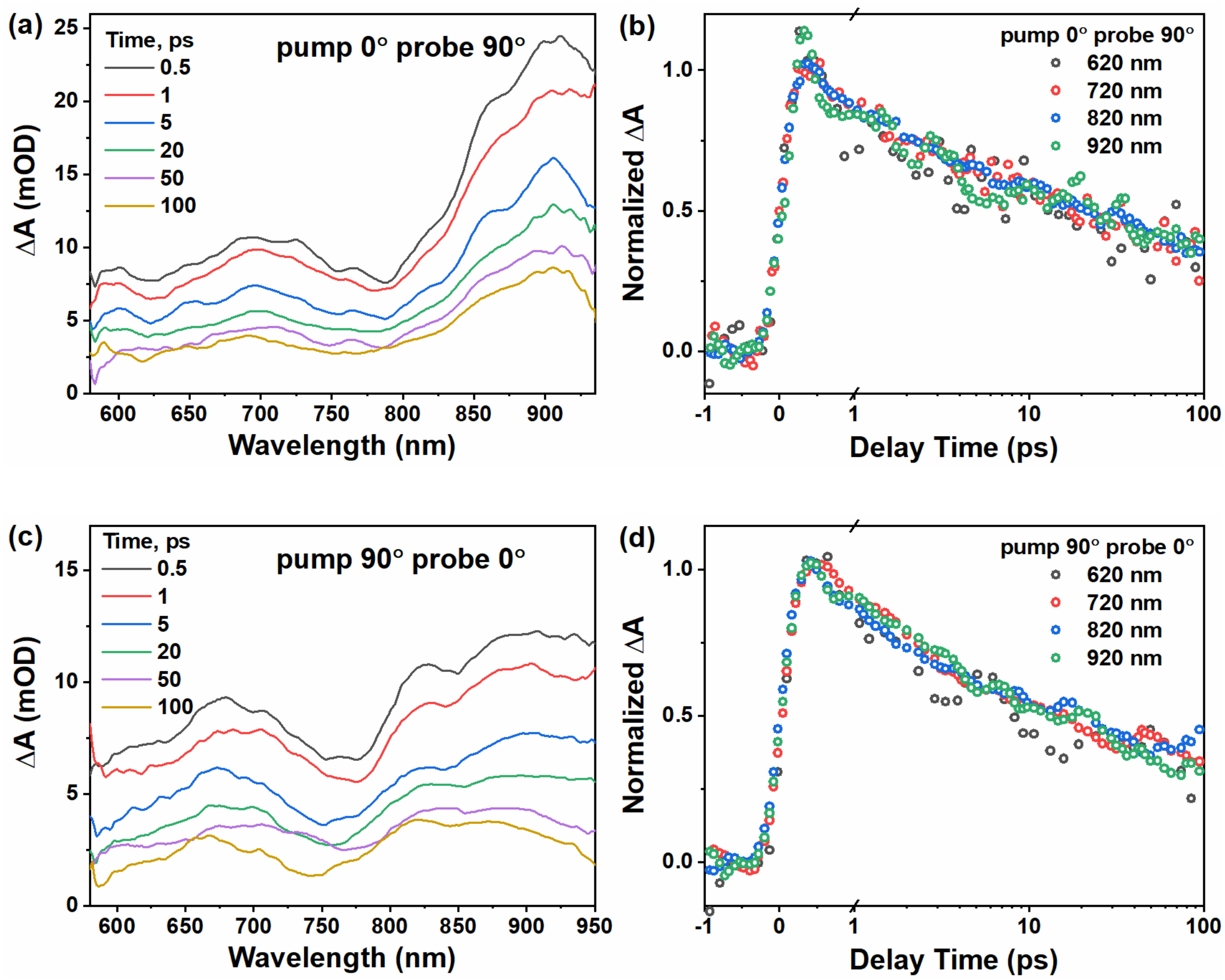

Figure S13. The TA spectra of SNH-FB crystal with different pump (400 nm) and probe polarizations: (a) pump 0°, probe 90°, (c) pump 90°, probe 0°. The angle represents the angle between the polarization of pump light or probe light and the long axis of the crystal. (b, d) The corresponding TA dynamics at different probe wavelengths.

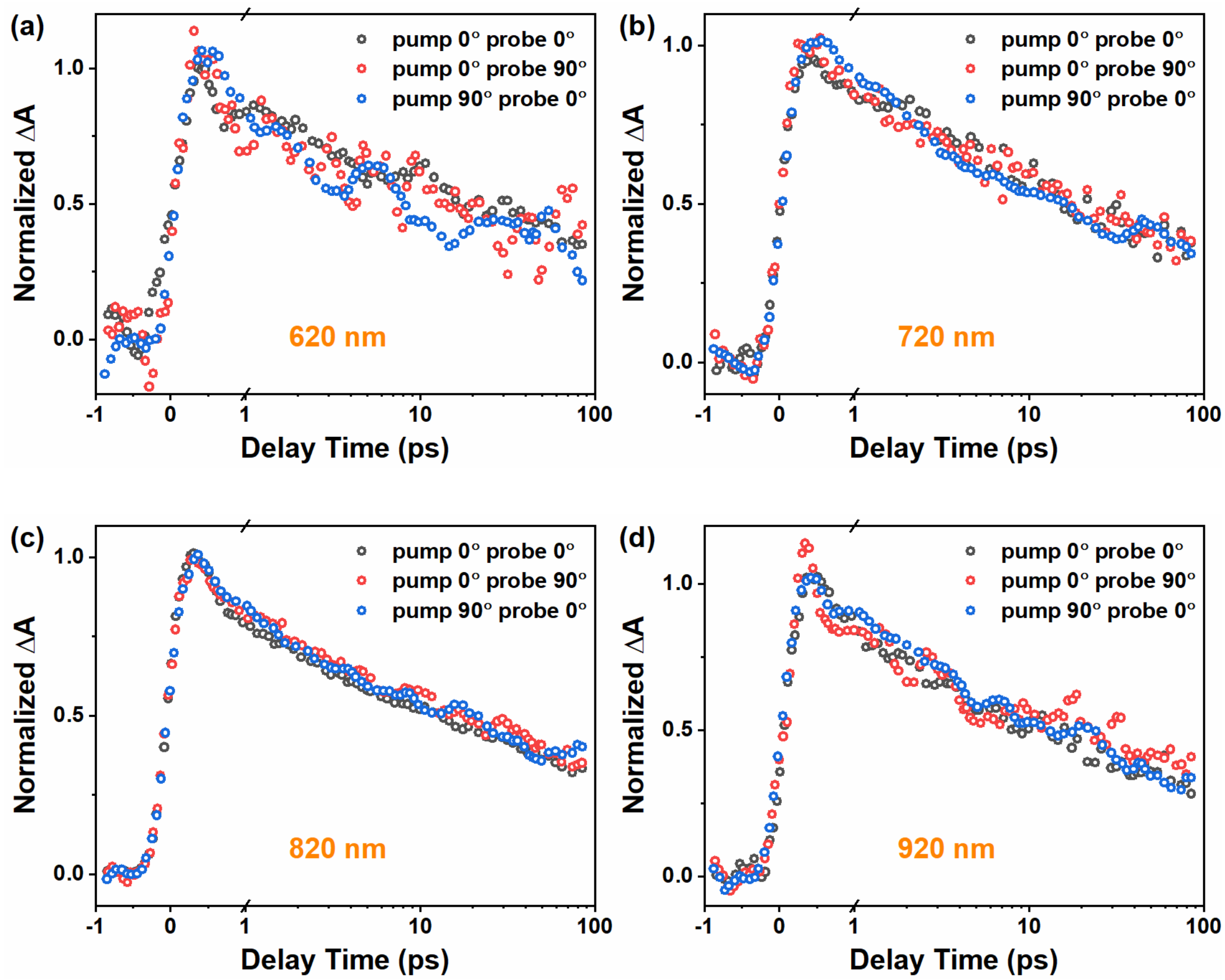

Figure S14. Polarization-dependent TA dynamics of SNH-FB crystal at different probe wavelengths: (a) 620 nm, (b) 720 nm, (c) 820 nm, (d) 920 nm.

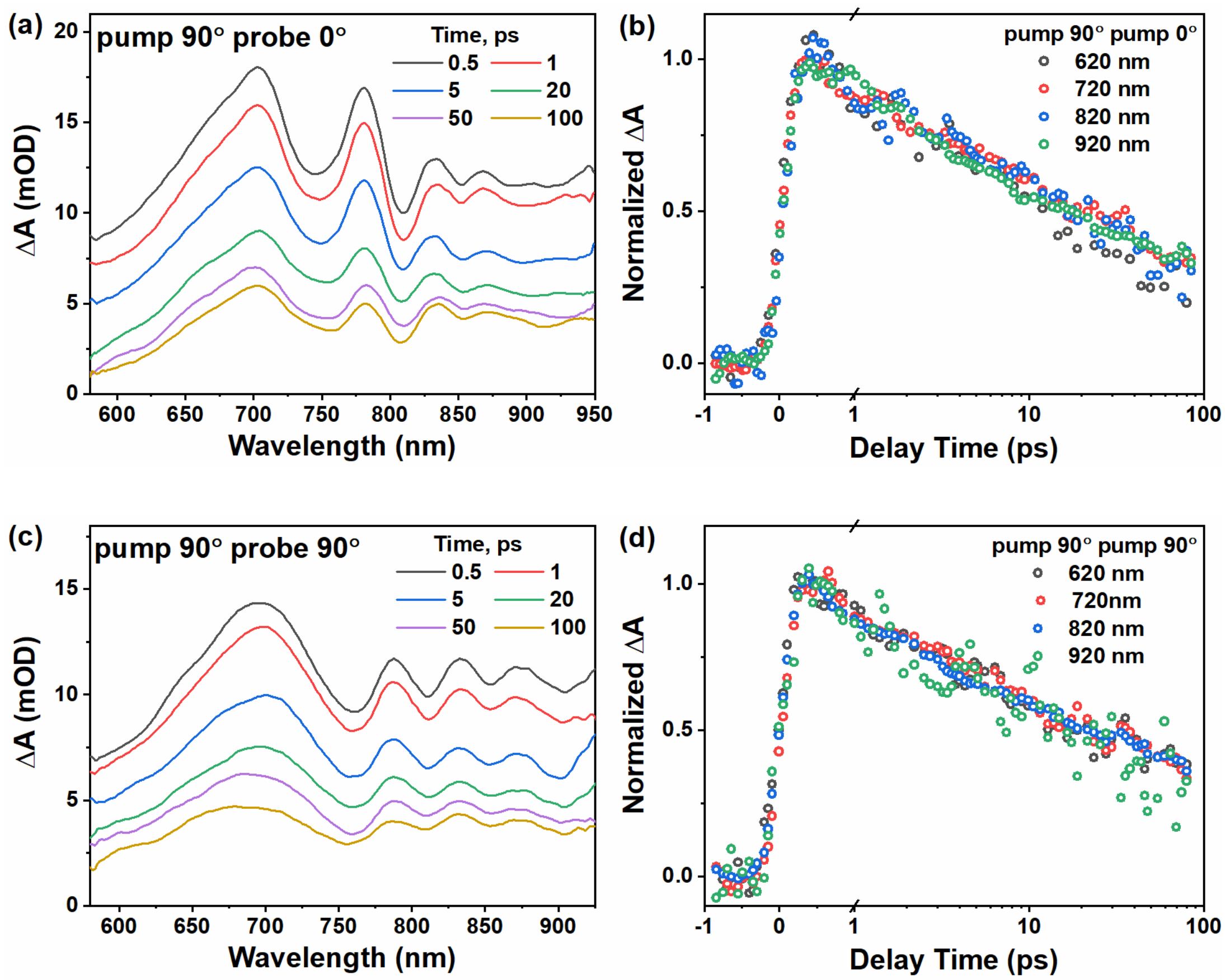

Figure S15. The TA spectra of SNEt-FB crystal with different pump (400 nm) and probe polarizations: (a) pump 90°, probe 0°, (c) pump 90°, probe 90°. The angle represents the angle between the polarization of pump light or probe light and the long axis of the crystal. (b, d) The corresponding TA dynamics at different probe wavelengths.

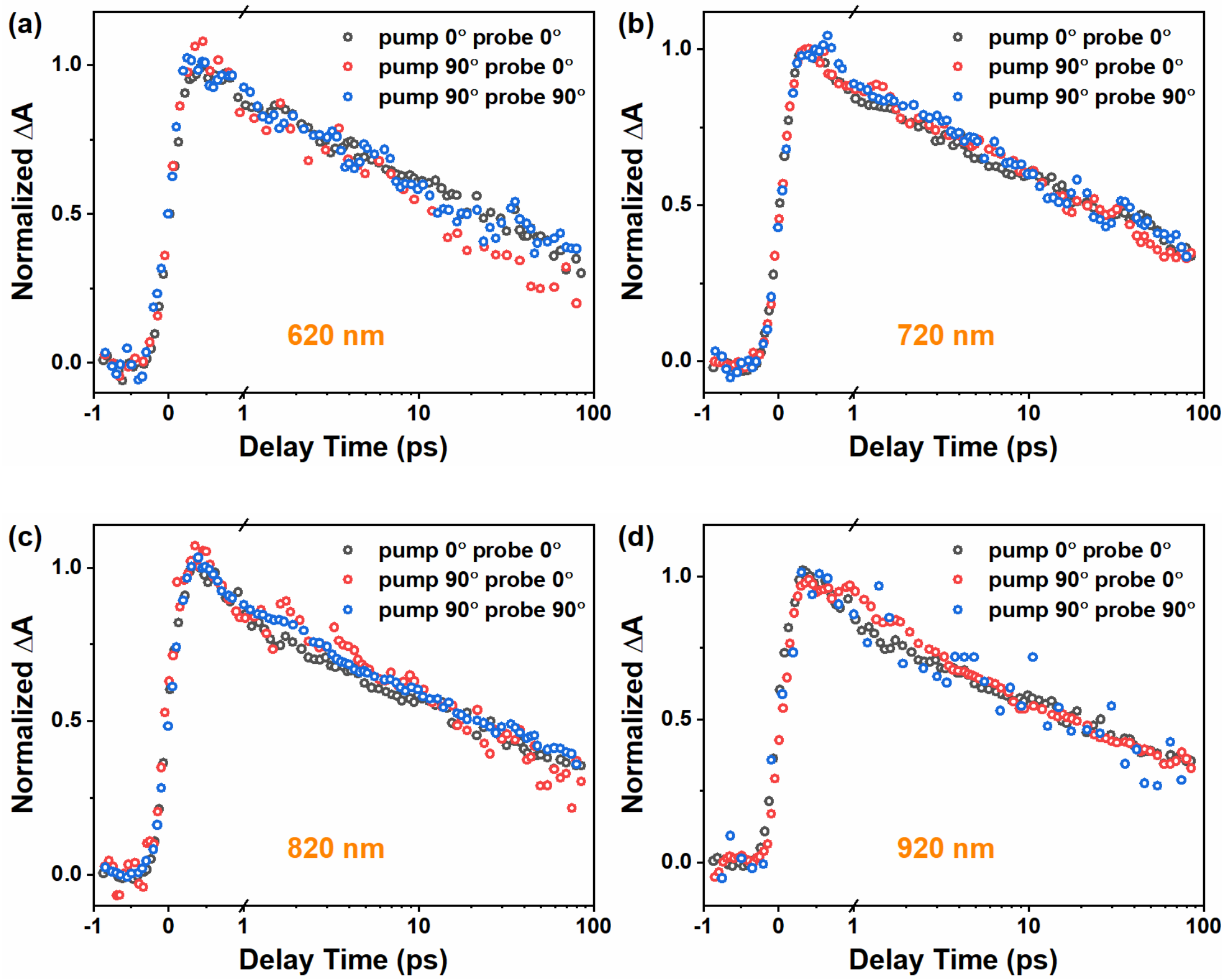

Figure S16. Polarization-dependent TA dynamics of SNEt-FB crystal at different probe wavelengths: (a) 620 nm, (b) 720 nm, (c) 820 nm, (d) 920 nm.

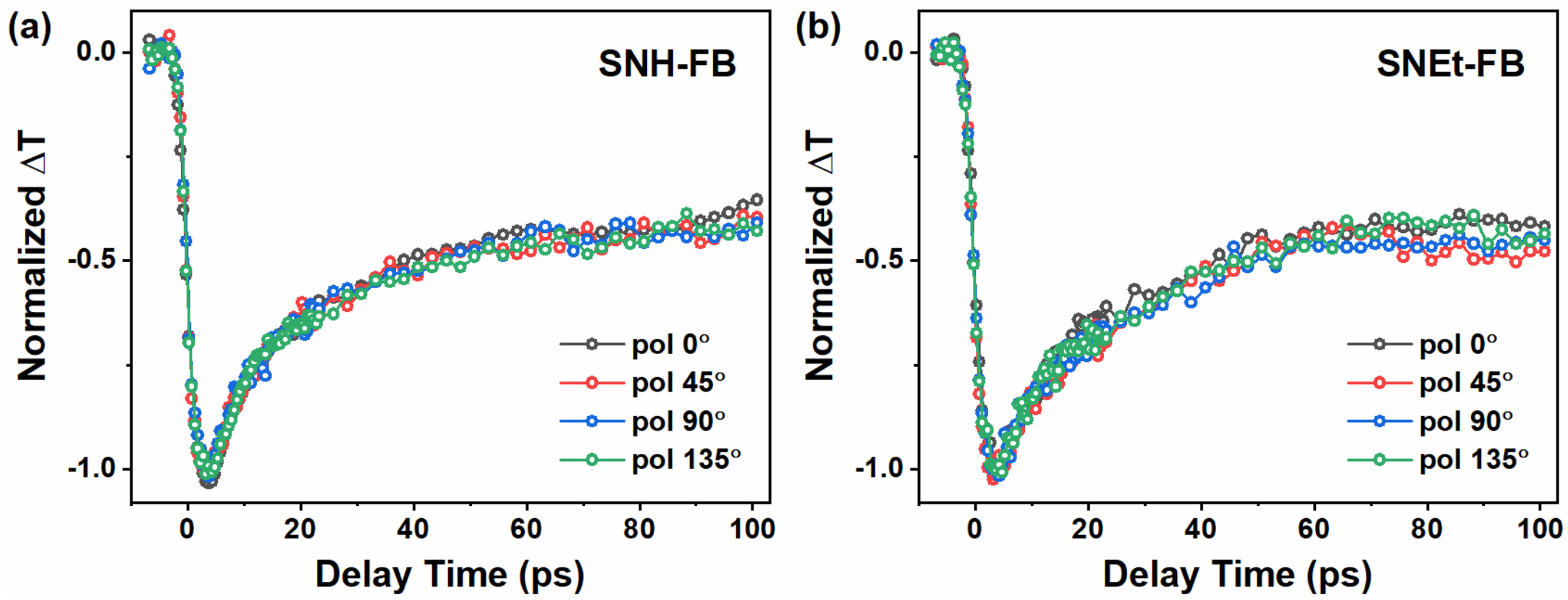

Figure S17. Normalized exciton dynamics of SNH-FB (a) and SNEt-FB (b) crystals under different probe polarizations obtained by TAM. The angle represents the angle between the probe light and the long axis of the crystal.

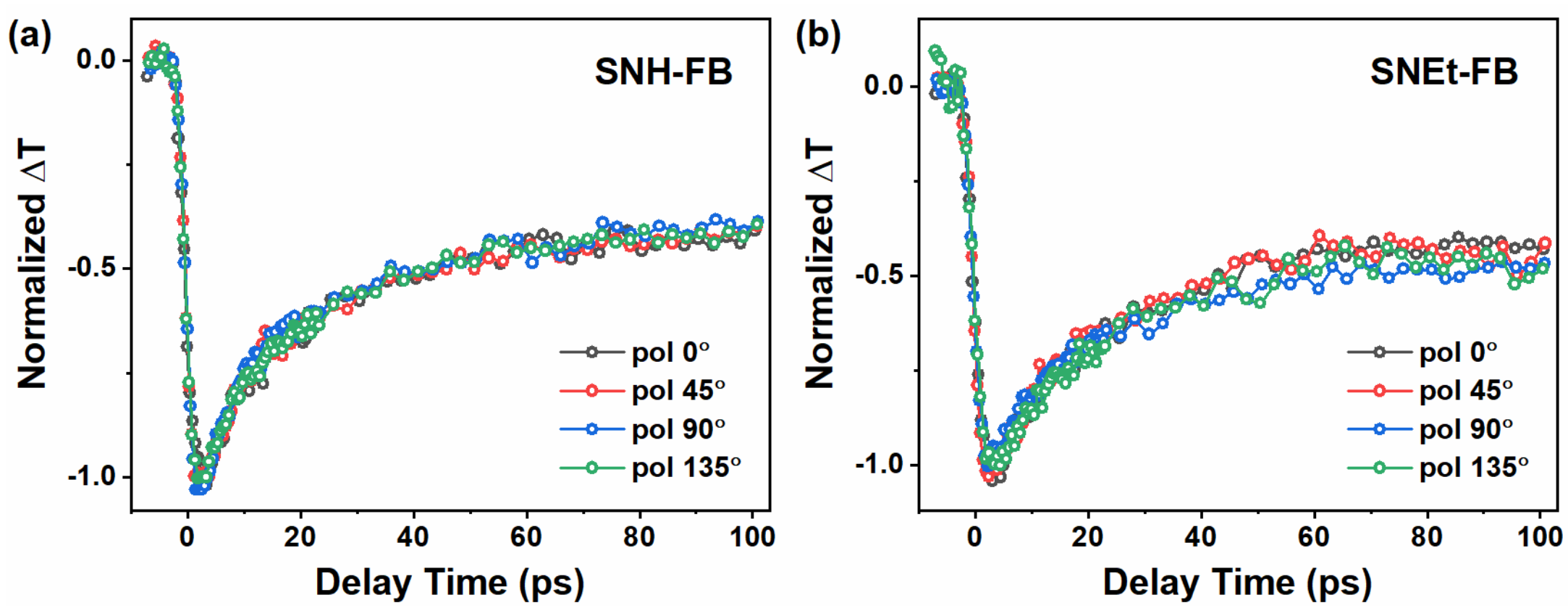

Figure S18. Normalized exciton dynamics of SNH-FB (a) and SNEt-FB (b) crystals under different pump polarizations obtained by TAM. The angle represents the angle between the pump light and the long axis of the crystal.

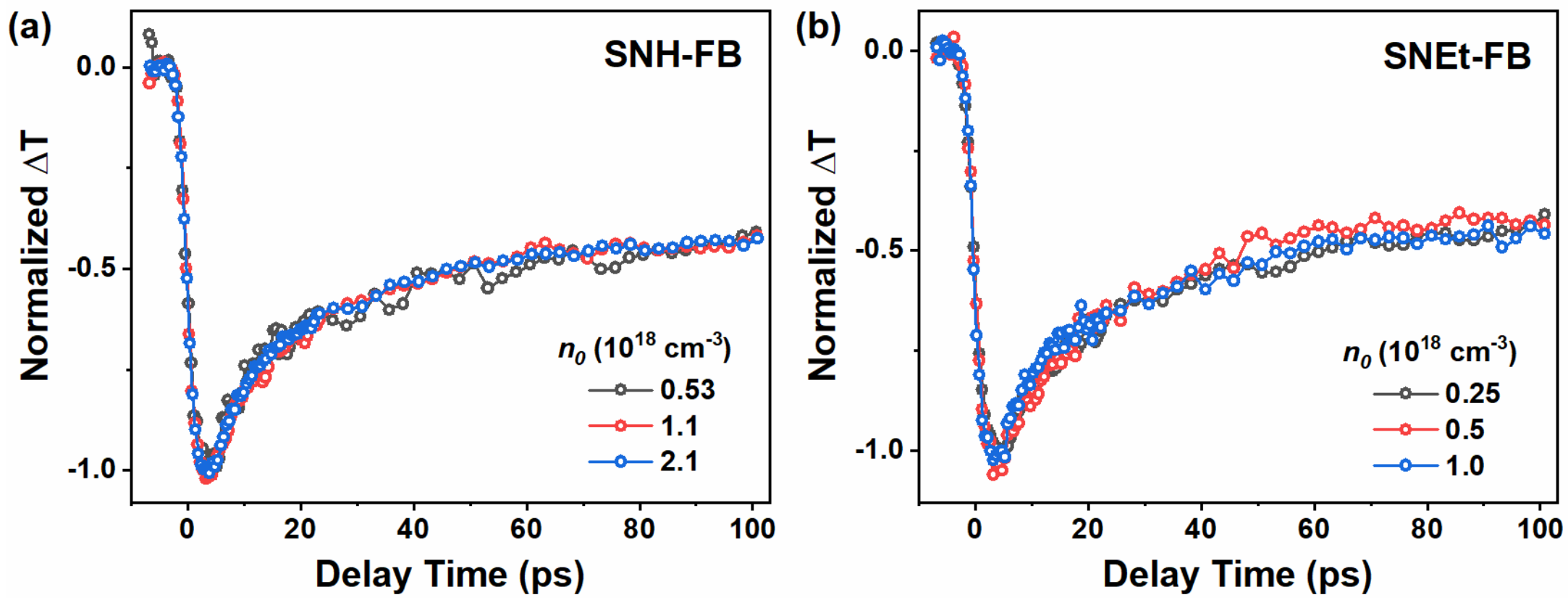

Figure S19. Normalized exciton dynamics of SNH-FB (a) and SNEt-FB (b) crystals at different initial exciton density n_0 obtained by TAM.

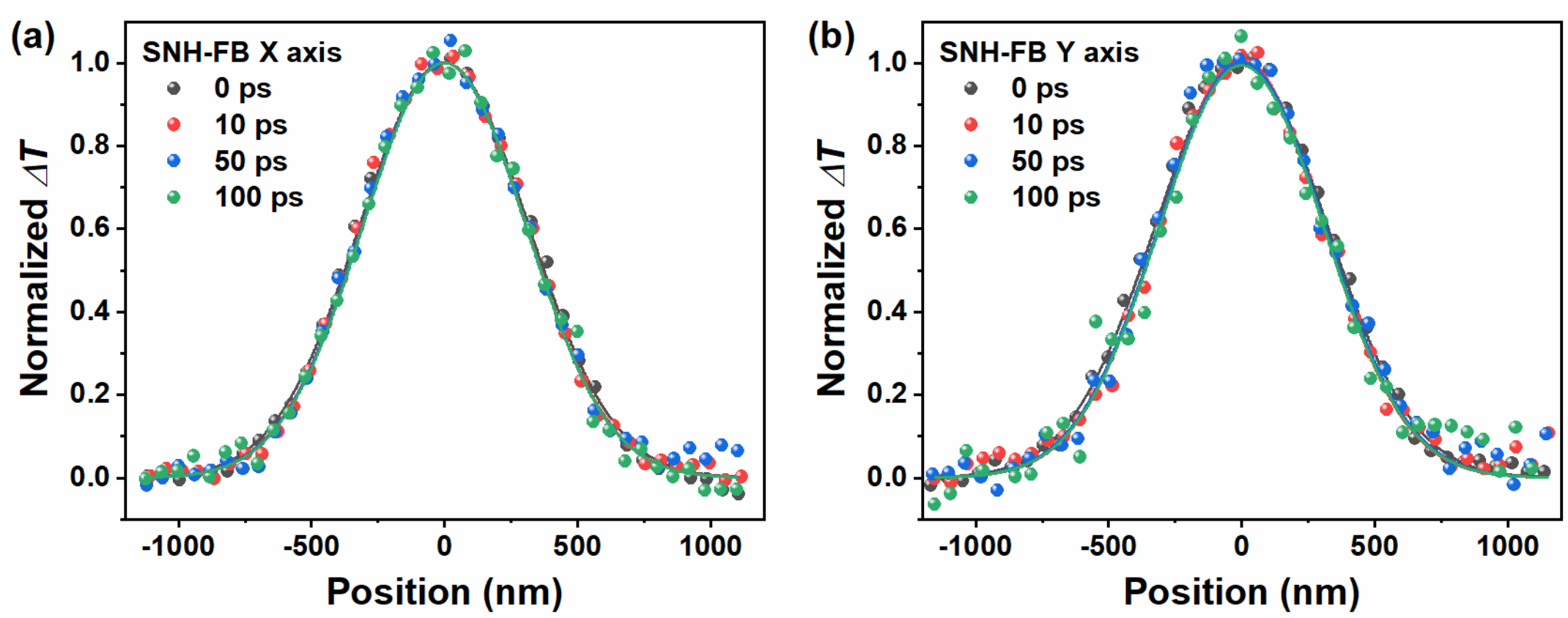

Figure S20. Cross sections of the TAM images of SNH-FB crystal fitted with Gaussian functions along the X and Y axes, with the maximum ΔT signal normalized.

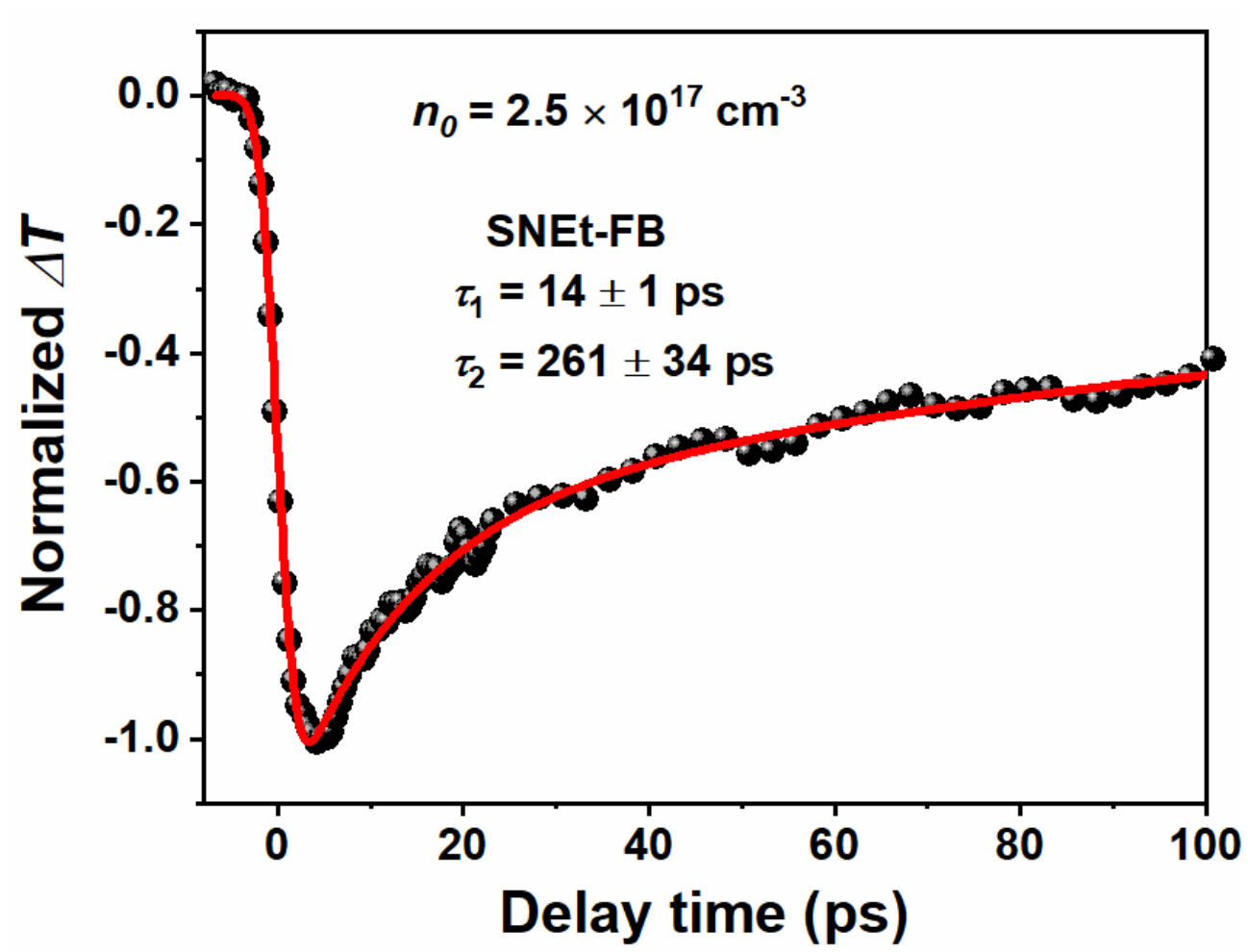

Figure S21. The exciton lifetimes of SNEt-FB crystal fitted from the TAM dynamics.

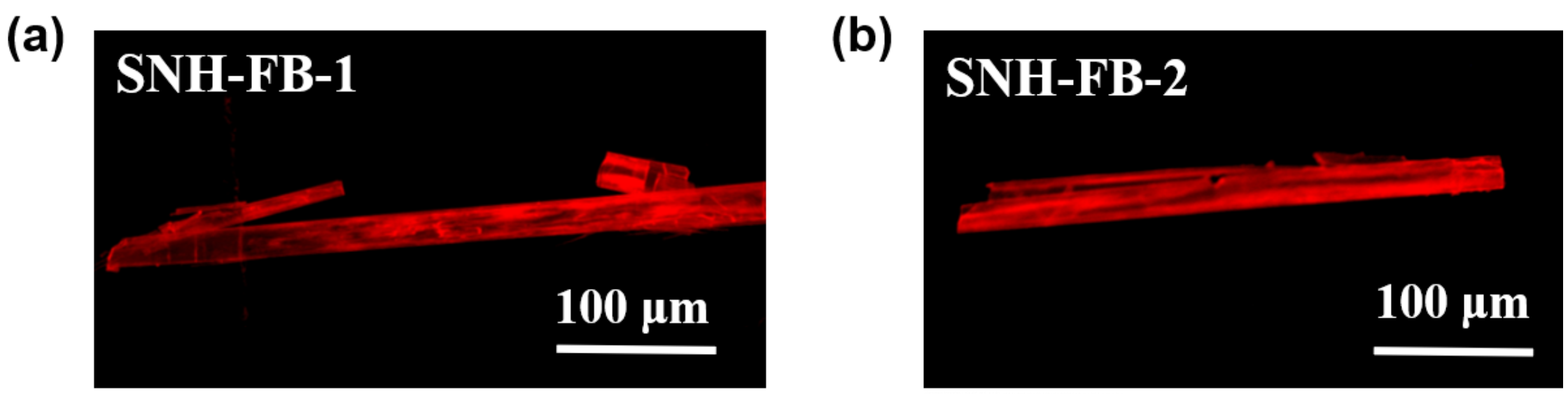

Figure S22. The PL microscopy imaging of two SNH-FB crystals for TAM imaging experiments.

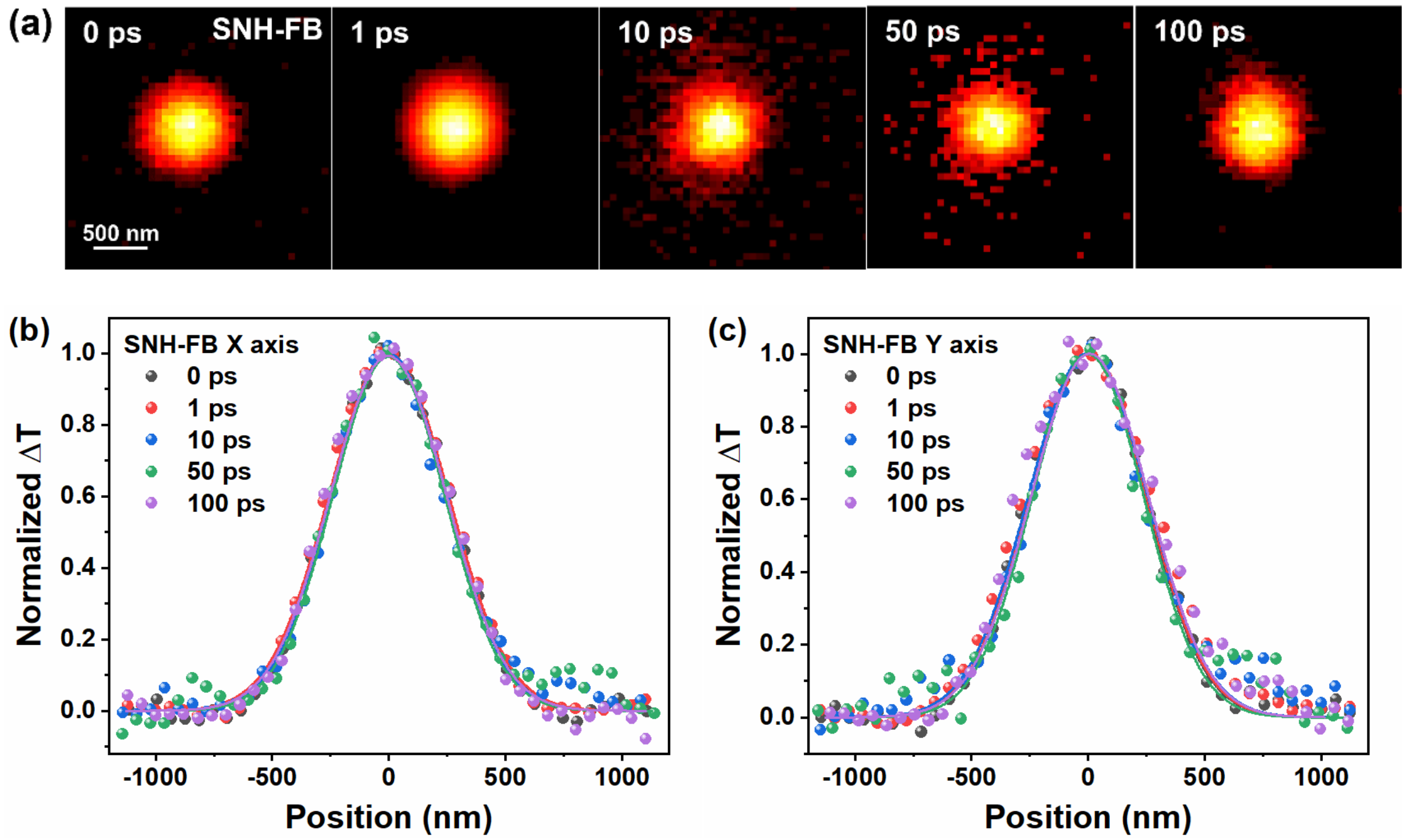

Figure S23. (a) Exciton diffusion imaging for SNH-FB-2 crystal ($n_0 = 1.2 \times 10^{18}$ cm^{-3}) obtained by TAM. The color scale represents the intensity of pump-induced differential transmission (ΔT) of the probe, and every image has been normalized by the peak value. (b,c) Cross sections of the TAM images of SNH-FB-2 crystal fitted with Gaussian functions along the X and Y axes, with the maximum ΔT signal normalized.

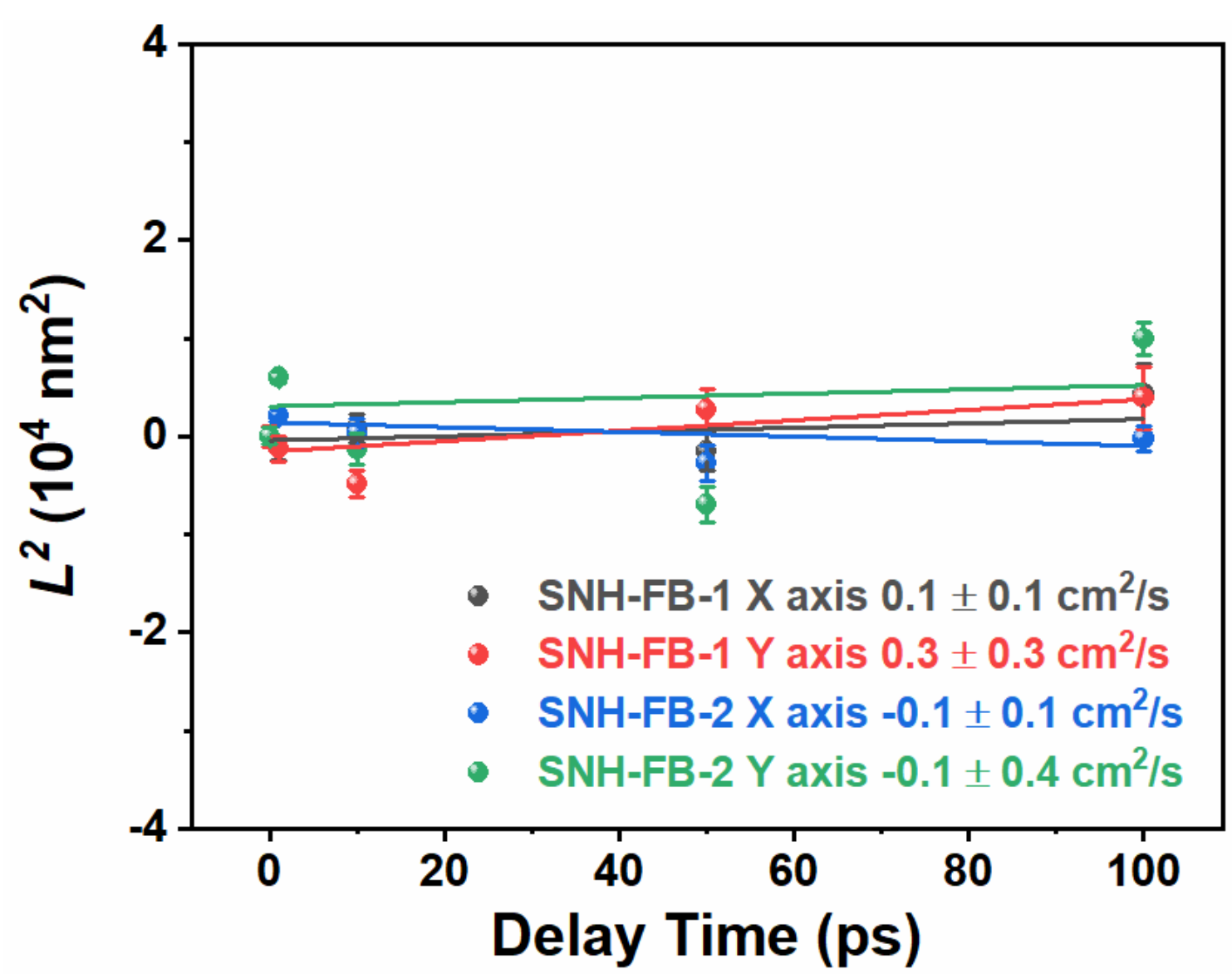

Figure S24. Time evolution and linear fitting of L^2 along the different axes for SNH-FB crystals. Error bars are the standard error estimated from the 2D Gaussian fitting to the spatial intensity distribution. The exciton diffusion constant D of different axis is indicated.

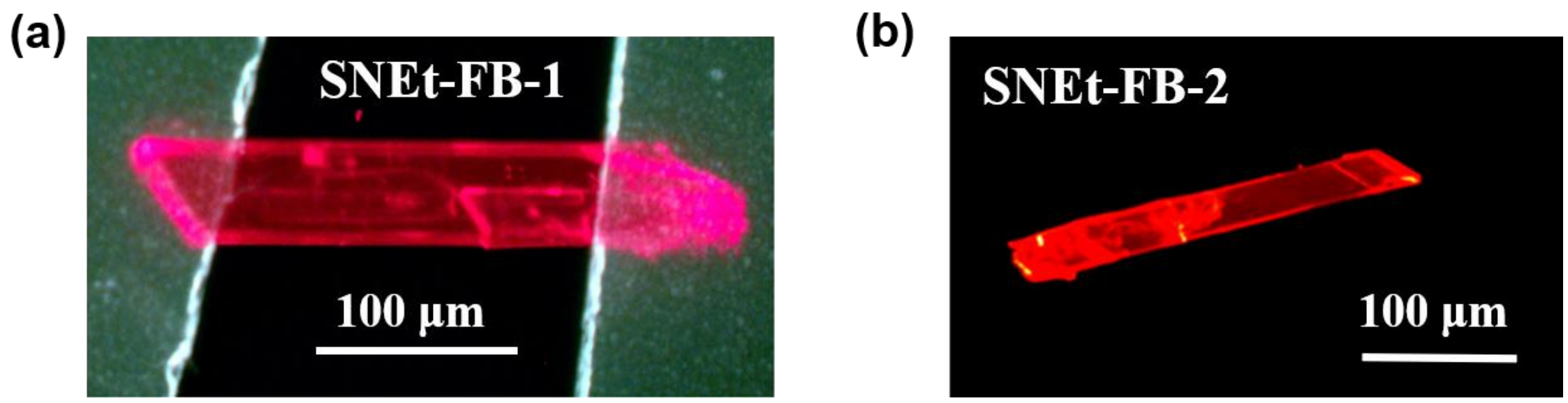

Figure S25. The PL microscopy imaging of two SNEt-FB crystals for TAM imaging experiments. Both of the crystals were aligned in the TAM apparatus according to the PL images. The green areas at both ends of the crystal in (a) are adhesive tapes for securing the crystal. The measurements were conducted at position devoid of any tape.

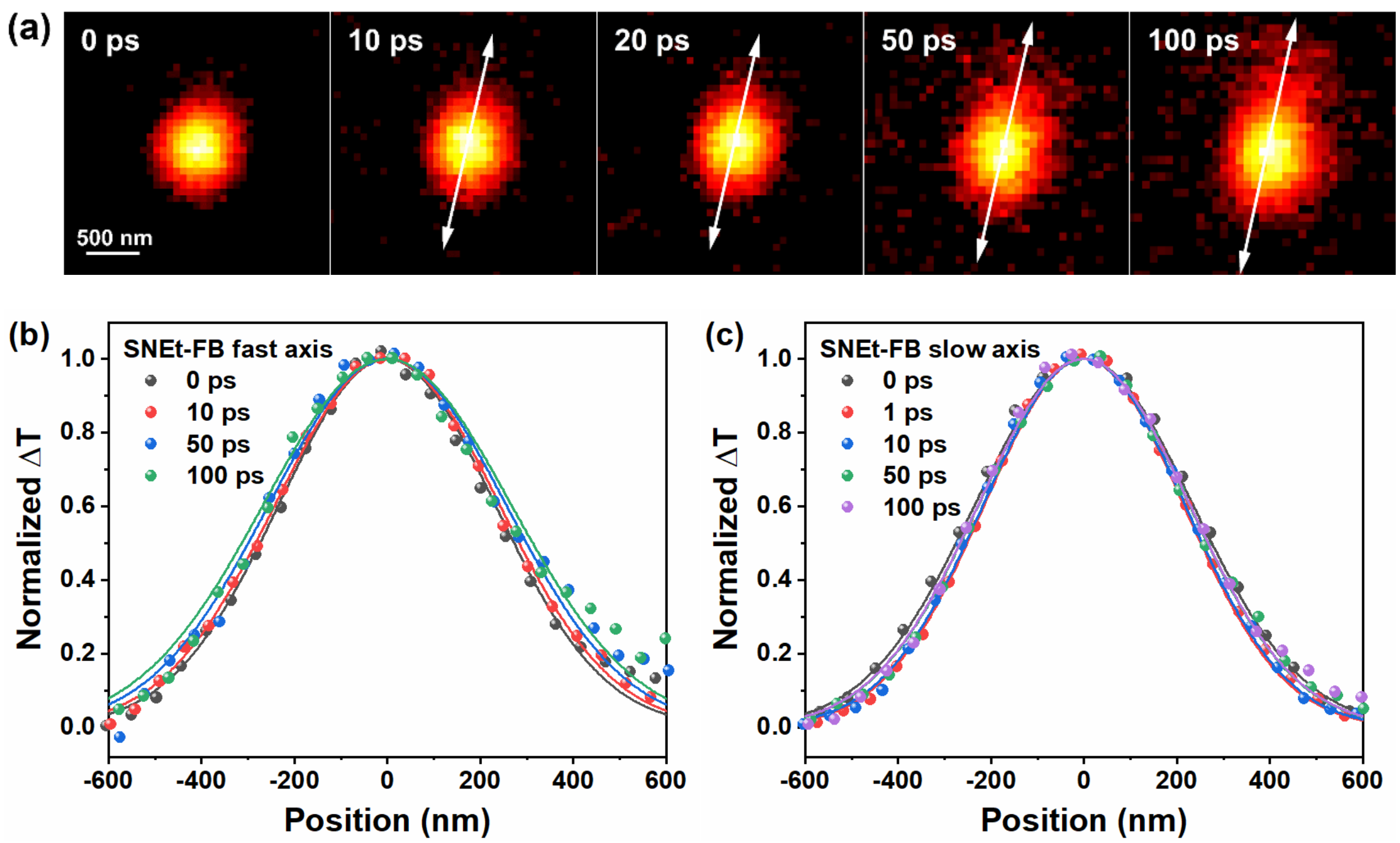

Figure S26. (a) Exciton diffusion imaging for SNEt-FB-2 crystal ($n_0 = 1.1 \times 10^{18}$ cm^{-3}) obtained by TAM. The color scale represents the intensity of pump-induced differential transmission (ΔT) of the probe, and every image has been normalized by the peak value. The white arrow in the TAM images indicates the fast transport axis. (b,c) Cross sections of the TAM images fitted with Gaussian functions along the white arrow and the direction perpendicular to the arrow, respectively, with the maximum ΔT signal normalized.

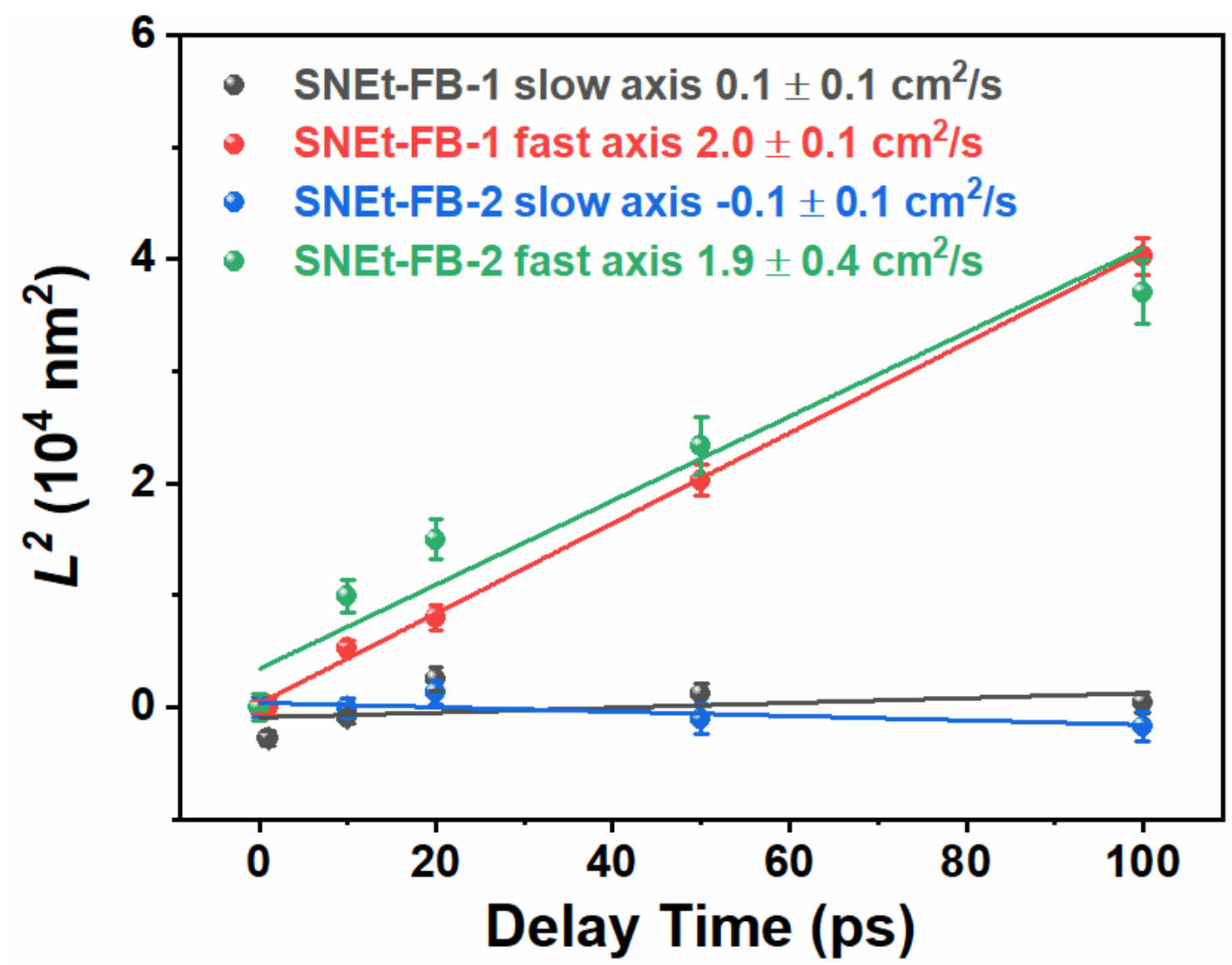

Figure S27. Time evolution and linear fitting of L^2 along the different axes for SNEt-FB crystals. Error bars are the standard error estimated from the 2D Gaussian fitting to the spatial intensity distribution. The exciton diffusion constant *D* of different axis is indicated.

Table S3. Comparison of excitons diffusion constants *D* and anisotropy ratios *k* with prior studies.

Sample	D_{fast} (cm^2/s)	D_{slow} (cm^2/s)	$k = D_{fast} / D_{slow}$
**SNEt-FB (current work)**	**2.0**	**0.1**	**20**
BCZ-Y[7]	2.0	0.9	2.2
PDHF[8]	2.56	1.57	1.63
Tetracene[9] (Triplet)	2.28×10^{-3}	1.35×10^{-3}<br>0.31×10^{-3}	1.67-7.35
Tetracene[10] (Singlet)	2.8	0.85	3.29
ANT[11] (theoretical)	3.3×10^{-3}	0.8×10^{-3}	4
a6T[11] (theoretical)	4.0×10^{-2}	6×10^{-3}	6.7
BF-e[12]	0.96	< 0.05	> 19.2
O-IDTBR[13] (theoretical)	8.3×10^{-2}	1.6×10^{-2}<br>1.4×10^{-2}	5-6

4. Theoretical calculation

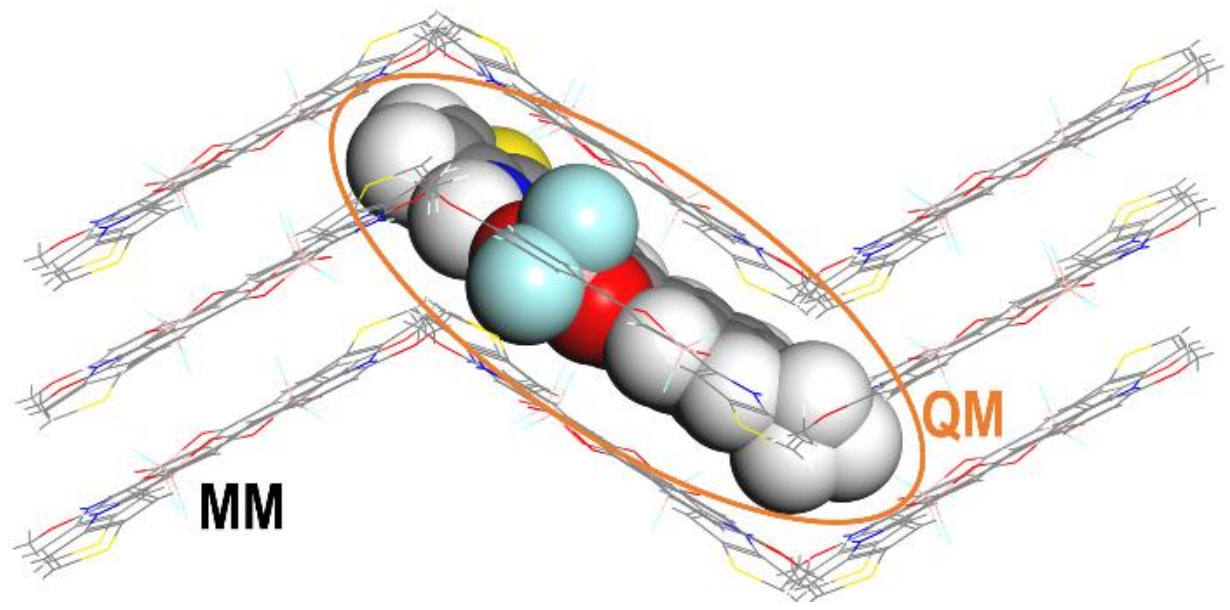

Figure S28. Setup of QM/MM Model (Taking SNH-FB as an Example).

Table S4. Theoretical calculated and experimental absorption and emission wavelengths for SNH-FB and SNEt-FB.

	λ_{abs}^{cal} / λ_{abs}^{exp} (nm)	λ_{emi}^{cal} / λ_{emi}^{exp} (nm)
SNH-FB	396.50/ 510.00	467.34/ 645.00
SNEt-FB	392.11/ 520.00	440.91/ 585.00

*The experimental absorption and emission wavelengths were measured in crystal.

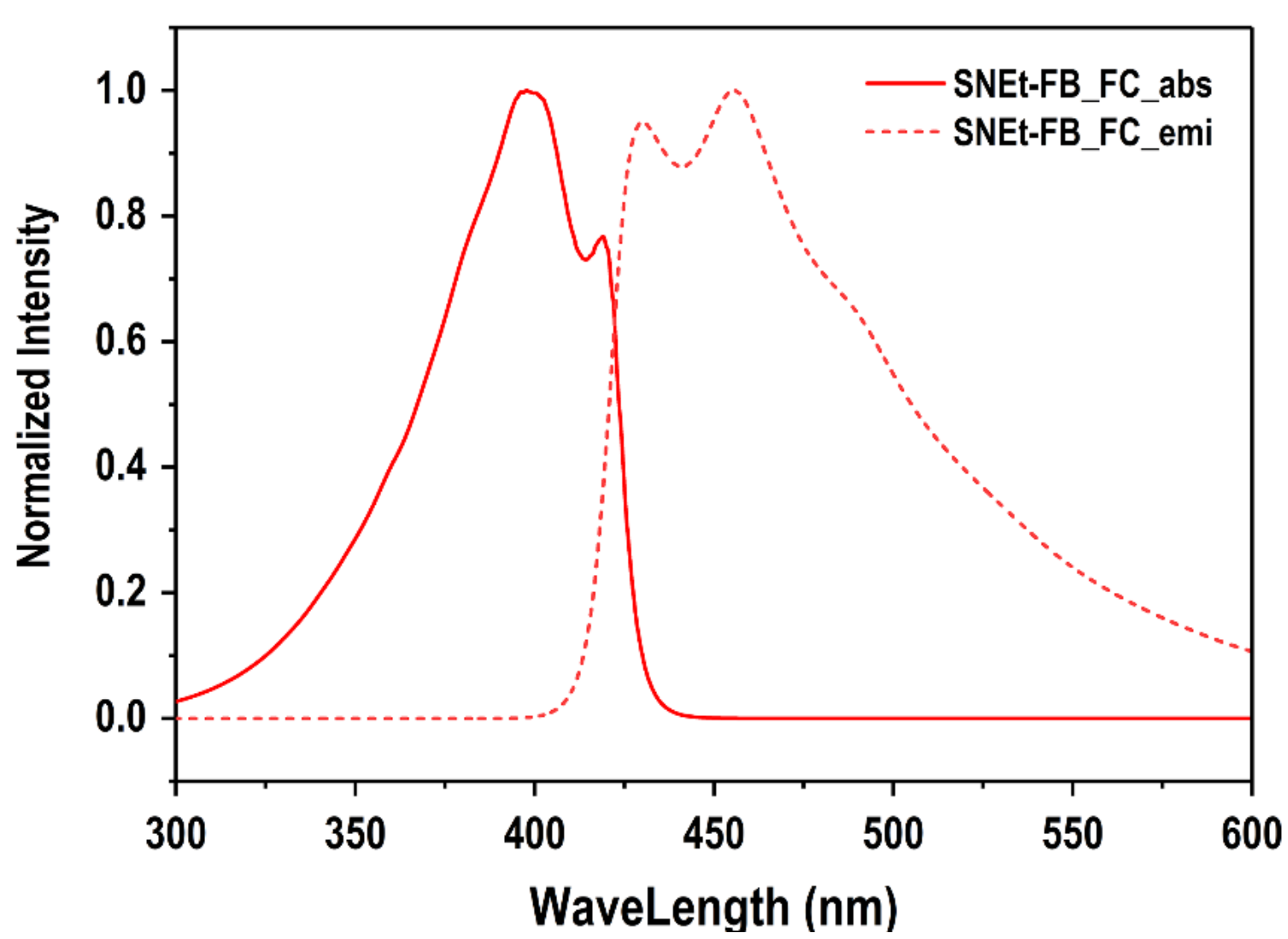

Figure S29. Theoretical absorption and emission spectra of SNEt-FB.

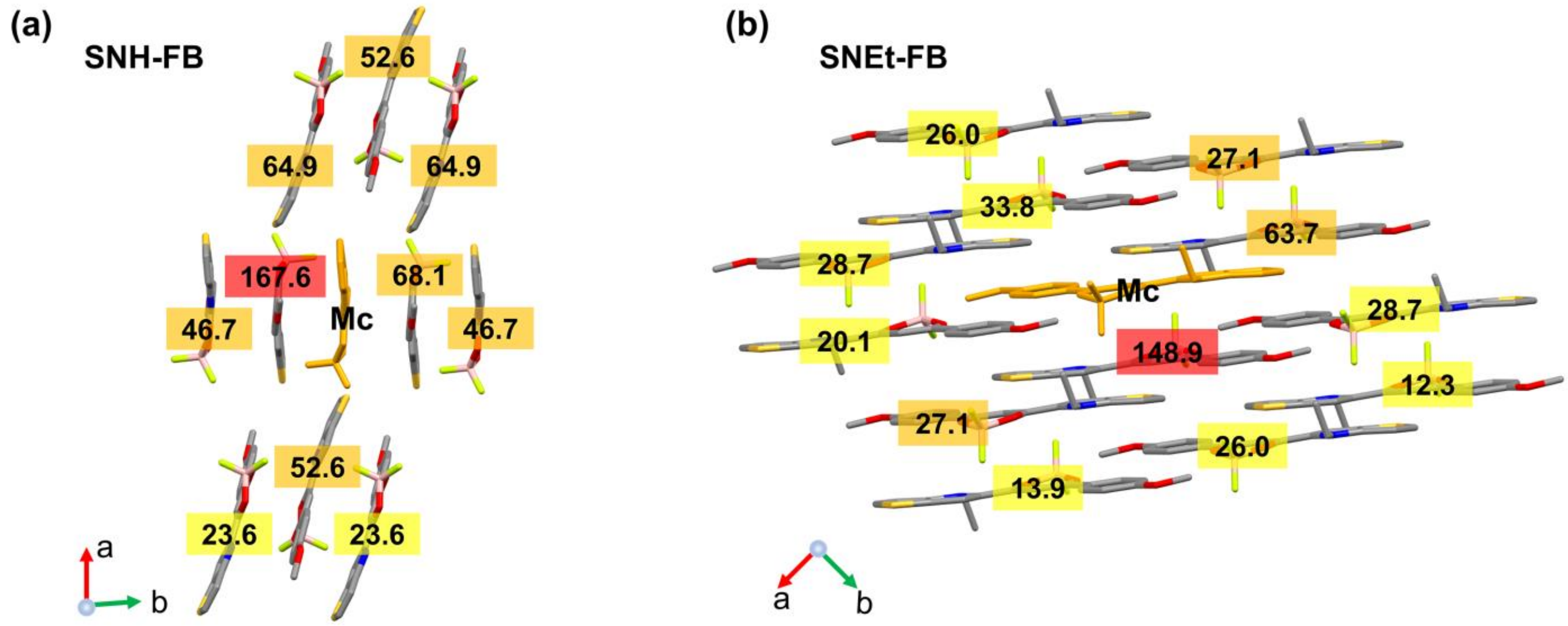

Figure S30. The excitonic couplings between the center molecule (Mc) and the neighboring molecules for (a) SNH-FB and (b) SNEt-FB, with hydrogen atoms omitted for clarity. The absolute value of excitonic couplings are marked (in unit of meV).

Table S5. Centroid distances (R), excitonic couplings (J), EET rates (k_{eet}) between selected dimers (D1−D26) in the SNH-FB crystal and the corresponding spectral overlap integral (I).

Dimers	R (Å)	J (meV)	I (eV^{-1})	k_{eet}(s^{-1})
D1	3.159	167.6	0.078	2.08×10^{13}
D2	5.544	68.1		3.44×10^{12}
D3	7.976	46.7		1.62×10^{12}
D4	7.976	46.7		1.62×10^{12}
D5	9.927	7.3		3.98×10^{10}
D6	9.927	7.3		3.98×10^{10}
D7	10.724	26.3		5.12×10^{11}
D8	10.724	26.3		5.12×10^{11}
D9	10.770	52.6		2.05×10^{12}
D10	10.770	52.6		2.05×10^{12}
D11	10.796	64.9		3.12×10^{12}
D12	10.796	64.9		3.12×10^{12}
D13	11.812	23.6		4.12×10^{11}
D14	11.812	23.6		4.12×10^{11}
D15	12.861	30.4		6.85×10^{11}
D16	12.861	30.4		6.85×10^{11}
D17	13.935	3.2		7.81×10^{9}
D18	13.935	3.2		7.81×10^{9}
D19	14.120	4.9		1.75×10^{10}
D20	14.120	4.9		1.75×10^{10}
D21	14.872	9.9		7.29×10^{10}
D22	14.872	9.9		7.29×10^{10}
D23	16.056	7.5		4.15×10^{10}
D24	16.056	7.5		4.15×10^{10}
D25	16.276	4.2		1.28×10^{10}
D26	16.276	4.2		1.28×10^{10}

Table S6. Centroid distances (R), excitonic couplings (J), EET rates (k_{eet}) between selected dimers (D1−D27) in the SNEt-FB crystal and the corresponding spectral overlap integral (I).

Dimers	R (Å)	J (meV)	I (eV^{-1})	k_{eet}(s^{-1})
D1	3.431	148.9		1.01×10^{14}
D2	6.934	33.8		5.20×10^{12}
D3	8.466	63.7		1.85×10^{13}
D4	8.946	27.1		3.35×10^{12}
D5	8.946	27.1		3.35×10^{12}
D6	8.985	26.0		3.08×10^{12}
D7	8.985	26.0		3.08×10^{12}
D8	9.135	44.4		8.99×10^{12}
D9	10.137	33.4		5.08×10^{12}
D10	10.414	20.1		1.84×10^{12}
D11	10.580	13.9		8.79×10^{11}
D12	10.998	28.7		3.76×10^{12}
D13	10.998	28.7		3.76×10^{12}
D14	11.120	50.9	0.478	1.18×10^{13}
D15	11.702	6.9		2.18×10^{11}
D16	11.788	49.6		1.12×10^{13}
D17	11.812	13.5		8.37×10^{11}
D18	11.929	3.3		4.95×10^{10}
D19	12.531	12.3		6.89×10^{11}
D20	12.770	15.3		1.07×10^{12}
D21	13.075	1.9		1.63×10^{10}
D22	13.204	28.9		3.82×10^{12}
D23	13.473	21.0		2.02×10^{12}
D24	13.669	4.2		8.14×10^{10}
D25	13.683	16.8		1.29×10^{12}
D26	13.925	35.4		5.72×10^{12}
D27	15.424	6.4		1.87×10^{11}

References

1. Li, M. *et al.* Triplet-triplet energy transfer inside the single organic nanocrystal revealed by microscopic time resolved spectroscopy. *J. Phys. Chem. C* **126**, 11033-11041 (2022).
2. Cesaria, M., Caricato, A. P., Martino, M. Realistic absorption coefficient of ultrathin films. *J. Opt.* **14**, 105701 (2012).
3. Hsu, C.-P. The electronic couplings in electron transferand excitation energy transfer. *Acc. Chem. Res.* **42**, 509-518 (2009).
4. You, Z. Q., Hsu, C. P. Theory and calculation for the electronic coupling in excitation energy transfer. *Int. J. Quantum Chem.* **114**, 102-115 (2014).
5. Shao, Y. *et al.* Advances in molecular quantum chemistry contained in the Q-Chem 4 program package. *Mol. Phys.* **113**, 184-215 (2014).
6. Niu, Y. *et al.* MOlecular MAterials Property Prediction Package (MOMAP) 1.0: a software package for predicting the luminescent properties and mobility of organic functional materials. *Mol. Phys.* **116**, 1078 (2018).
7. Li, M. *et al.* The rapid transport of excitons in organic crystals can be regulated by the molecular packing form. *J. Phys. Chem. Lett* **16**, 955-962 (2025).
8. Cai, J. *et al.* Uniform conjugated polymer rectangular platelets exhibiting long-range exciton diffusion. *Nat. Mater.* **24**, 1985-1992 (2025).
9. Akselrod, G. M. *et al.* Visualization of exciton transport in ordered and disordered molecular solids. *Nat. Commun.* **5**, 3646 (2014).
10. Wan, Y. *et al.* Cooperative singlet and triplet exciton transport in tetracene crystals visualized by ultrafast microscopy. *Nat. Chem.* **7**, 785-792 (2015).
11. Giannini, S. *et al.* Exciton transport in molecular organic semiconductors boosted by transient quantum delocalization. *Nat. Commun.* **13**, 2755 (2022).
12. Baronas, P. *et al.* Exciton diffusion in bifluorene single crystals studied by light induced transient grating technique. *Appl. Phys. Lett.* **112**, 033302 (2018).
13. Stojanovic, L., Giannini, S., Blumberger, J. Exciton transport in the nonfullerene acceptor O-IDTBR from nonadiabatic molecular dynamics. *J. Chem. Theory Comput.* **20**, 6241-6252 (2024).
